# Defect Engineering in Solution-Processed Polycrystalline SnSe Leads to High Thermoelectric Performance

**DOI:** 10.1021/acsnano.1c06720

**Published:** 2021-09-22

**Authors:** Yu Liu, Mariano Calcabrini, Yuan Yu, Seungho Lee, Cheng Chang, Jérémy David, Tanmoy Ghosh, Maria Chiara Spadaro, Chenyang Xie, Oana Cojocaru-Mirédin, Jordi Arbiol, Maria Ibáñez

**Affiliations:** † IST Austria, Am Campus 1, 3400 Klosterneuburg, Austria; ‡ RWTH Aachen, I. Physikalisches Institut (IA), Sommerfeldstraße 14, 52074 Aachen, Germany; § Catalan Institute of Nanoscience and Nanotechnology (ICN2), CSIC and BIST, Campus UAB, Bellaterra, 08193 Barcelona, Catalonia, Spain; ∥ Department of Physics, INTE & Barcelona Multiscale Res. Center, Universitat Politècnica de Catalunya, Avda. Eduard Maristany 16, 08930 Barcelona, Catalunya, Spain; ⊥ ICREA, Pg. Lluis Companys 23, 08010 Barcelona, Catalonia, Spain

**Keywords:** tin selenide, nanocomposite, grain growth, Zener pinning, thermoelectricity, annealing, solution processing

## Abstract

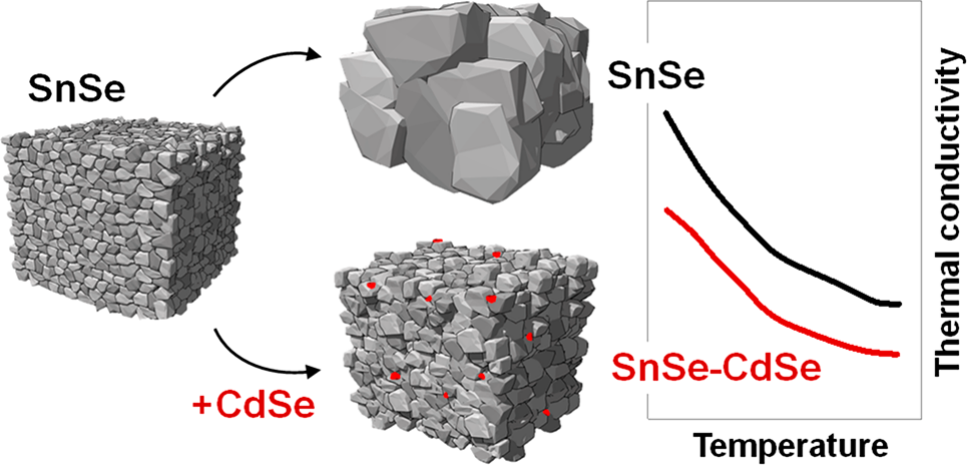

SnSe has emerged as one of the most promising materials for thermoelectric energy conversion due to its extraordinary performance in its single-crystal form and its low-cost constituent elements. However, to achieve an economic impact, the polycrystalline counterpart needs to replicate the performance of the single crystal. Herein, we optimize the thermoelectric performance of polycrystalline SnSe produced by consolidating solution-processed and surface-engineered SnSe particles. In particular, the SnSe particles are coated with CdSe molecular complexes that crystallize during the sintering process, forming CdSe nanoparticles. The presence of CdSe nanoparticles inhibits SnSe grain growth during the consolidation step due to Zener pinning, yielding a material with a high density of grain boundaries. Moreover, the resulting SnSe–CdSe nanocomposites present a large number of defects at different length scales, which significantly reduce the thermal conductivity. The produced SnSe–CdSe nanocomposites exhibit thermoelectric figures of merit up to 2.2 at 786 K, which is among the highest reported for solution-processed SnSe.

## Introduction

Materials able to reversibly convert heat into electricity, i.e., thermoelectric materials, require high electrical conductivity (σ), high Seebeck coefficient (*S*), and low thermal conductivity (κ). The optimization of these three strongly interrelated properties involves tuning the electronic structure of the material and the charge and phonon scattering mechanisms.^1−4^

Zhao et al. discovered in 2014 that SnSe has an outstanding thermoelectric performance, originating an outburst of research on the material.^5−12^ The highest figure of merit (*zT* = σ*S*^2^*T*κ^–1^) obtained to date in *p*-type SnSe is ∼2.6 at 923 K along the *b*-axis in pristine SnSe single crystals^13^ and ∼2.8 at 773 K along the *a*-axis in Br-doped *n*-type SnSe single crystals.^14^ However, the high cost and stagnant production of single crystals, together with their poor mechanical properties, limit the large-scale use of SnSe in thermoelectric devices.^15^ A potential solution is shifting to polycrystalline SnSe-based materials. The problem is that polycrystalline SnSe suffers from lower thermoelectric performance due to oxidation leading to higher thermal conductivities, partial loss of anisotropy diminishing electrical conductivity, and imprecise control of the doping level.^16,17^

Different approaches to overcome these limitations include chemical reduction of oxide species,^16,17^ liquid-phase compaction^18^ and hot deformation processes to promote texture,^19^ and doping control with different alkali (K, Na, and Li)^20−22^ and transition (Ag, Cu, Zn, and Cd) metals.^23−29^ Additionally, approaches have been scrutinized to further enhance the polycrystalline materials’ performance, such as alloying with SnS,^30^ Pb,^31^ and Ge^32^ and the introduction of different nanofeatures such as nanoporous or nanoprecipitates (i.e., InSe_*y*_,^33^ AgSnSe_2_,^34^ PbSe,^35^ and Ag_8_SnSe_6_^36^).

Herein we report a simple and scalable synthesis route to produce SnSe–CdSe nanocomposites based on the aqueous synthesis of SnSe particles and their surface treatment with CdSe molecular complexes. Such surface treatment allows engineering of the material microstructure by promoting defect formation at all length scales. In particular, during the processing, CdSe complexes crystallize, forming CdSe nanoparticles (NPs) at the surface of SnSe particles. CdSe NPs hinders grain growth during consolidation yielding a material with a high density of multiscale defects (point defects, dislocations, planar defects, and nanostructures). The presence of CdSe NPs together with the significantly higher defect content results in a reduction of the thermal conductivity by 2-fold with respect to bare SnSe produced by untreated SnSe particles. Overall, the strategy presented here produces inexpensive and highly stable polycrystalline SnSe with a *zT* of ca. 2.2 at 786 K.

## Results and Discussion

**Figure 1:**
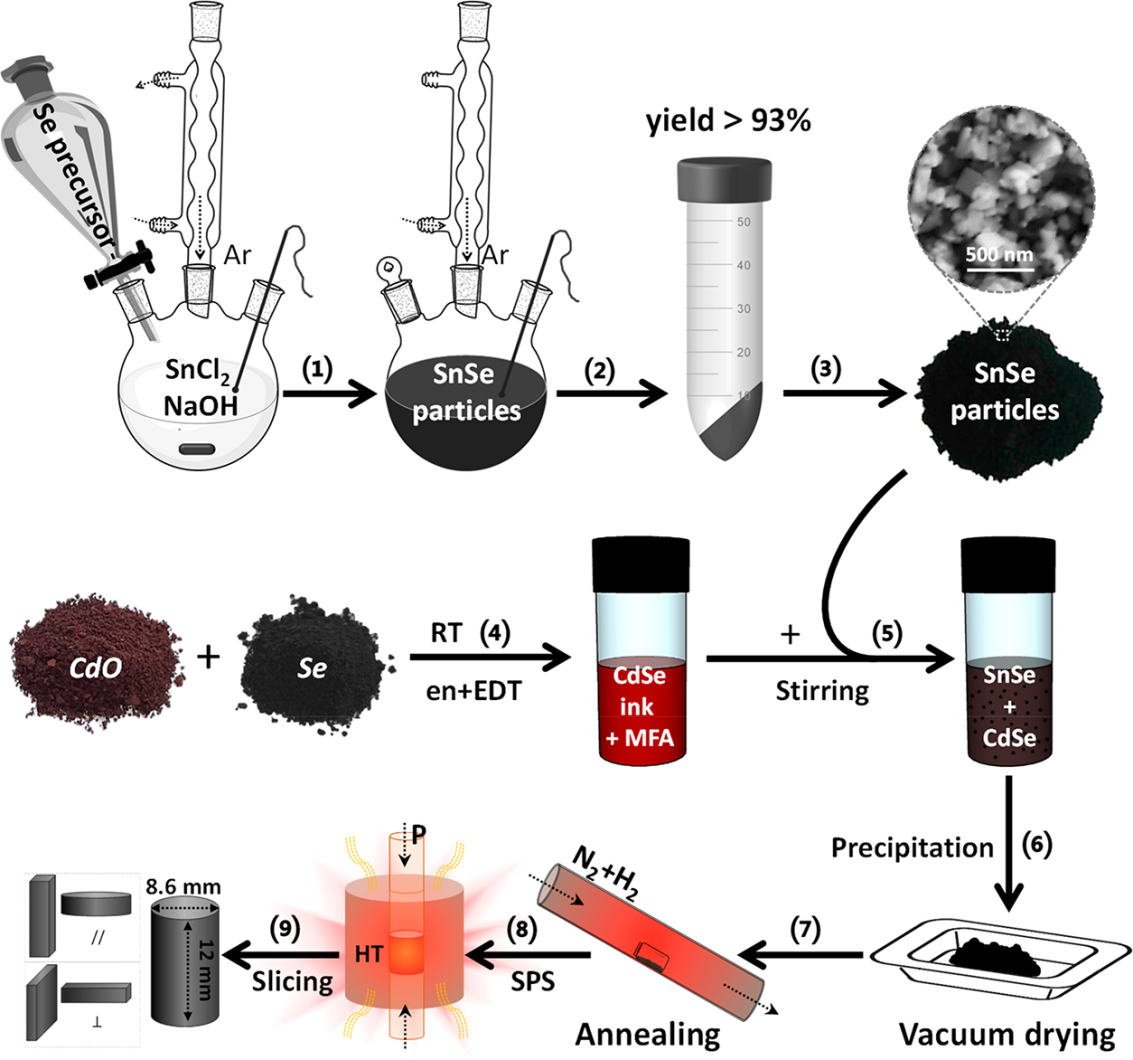
Scheme of the fabrication process of SnSe–CdSe nanocomposites. (1–3) Aqueous synthesis and purification of SnSe particles; (4) preparation of CdSe molecular solution (CdSe ink); (5) blending the CdSe ink with SnSe particles; (6) purification of the CdSe surface-treated SnSe particles and SnSe–CdSe powder vacuum drying; (7) annealing; (8) spark plasma sintering (SPS) for producing cylinders (⌀ = 8.6 mm × *h* = 12 mm); and (9) slicing for transport measurements.

SnSe particles were prepared in water, using Se powder and tin chloride hydrate as precursors.^37^ The obtained SnSe particles were purified to remove unbound ionic impurities by washing them with water and ethanol. To produce dense SnSe polycrystalline materials, the purified particles were then dried under vacuum, annealed in forming gas (95% N_2_ + 5% H_2_)^16,17^ at 500 °C, and consolidated into cylindrical pellets through spark plasma sintering (experimental details can be found in the “Methods” section). In the case of SnSe–CdSe nanocomposites, SnSe particles were mixed with CdSe molecular complexes (*x* mol %; *x* = 1, 2, 3, and 4) in *N*-methyl formamide for 48 h before annealing. The CdSe molecular solution was prepared by dissolving stoichiometric amounts of CdO and Se powder in a thiol–amine mixture (1,2-ethaneditiol, en; ethylenediamine, EDT) at room temperature in an inert atmosphere (Figure 1).^38^ On the basis of previous studies of hydrazinium-based CdSe solutions, we hypothesize that the molecular solute is composed of a variety of chalcogenidocadmates such as (Cd_2_Se_3_)_*n*_^2*n*–^ or CdSe_2_^2–^.^39−42^ The adsorption of CdSe species on the SnSe surface was verified by tracking the color change of the solution. The vivid orange color of the CdSe solution changed to a slightly yellow color after SnSe particles were introduced in the solution (Figure S1). Finally, CdSe surface-treated SnSe particles were precipitated from solution, washed twice with acetone, and dried under vacuum for further processing into cylindrical pellets. The complete material fabrication process is illustrated in Figure 1.

**Figure 2:**
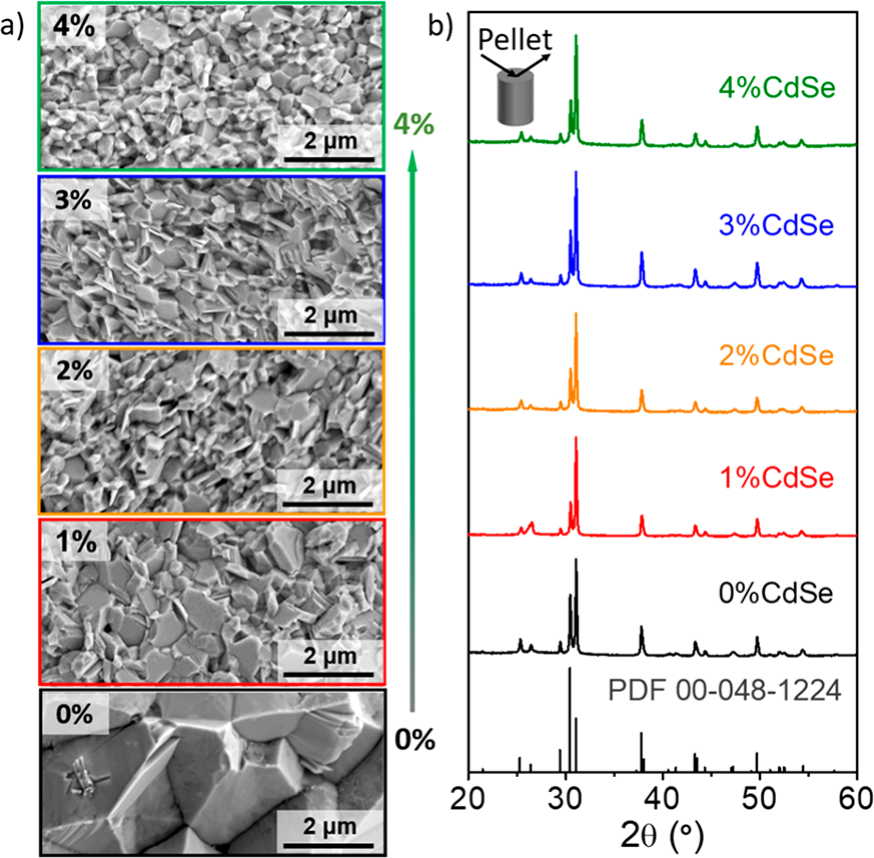
(a) Representative SEM images of the pellets obtained from SnSe-*x*%CdSe (*x* = 0, 1, 2, 3, and 4) particles; (b) the corresponding XRD patterns in the direction perpendicular to the pressing axis including the SnSe reference pattern (Powder Diffraction File (PDF) no. 00–048–1224, Joint Committee on Powder Diffraction Standards (JCPDS)).

Figure 2a shows scanning electron microscopy (SEM) images of the pellets obtained from SnSe-*x*%CdSe particles. In the presence of CdSe, the sintered materials present smaller crystal domains than bare SnSe despite having all similar densities (Table S1). Lower CdSe content than the estimated to coat the whole SnSe particles (1 mol %) resulted in larger grain sizes, but still smaller than that without any surface treatment. Above 2 mol %, the final grain size of all the pellets analyzed is practically the same.

X-ray diffraction (XRD) data showed no evidence of the presence of secondary phases in any nanocomposite (Figures 2b and S2). The grain size of the nanocomposites is stable and hardly changes after the consolidation step, as observed in the structural analysis of the pellets subjected to further thermal treatment such as heating and cooling cycles from room temperature to 823 K carried out during the transport measurements (Figure S3). These results evidence the high stability of the grain size.

**Figure 3:**
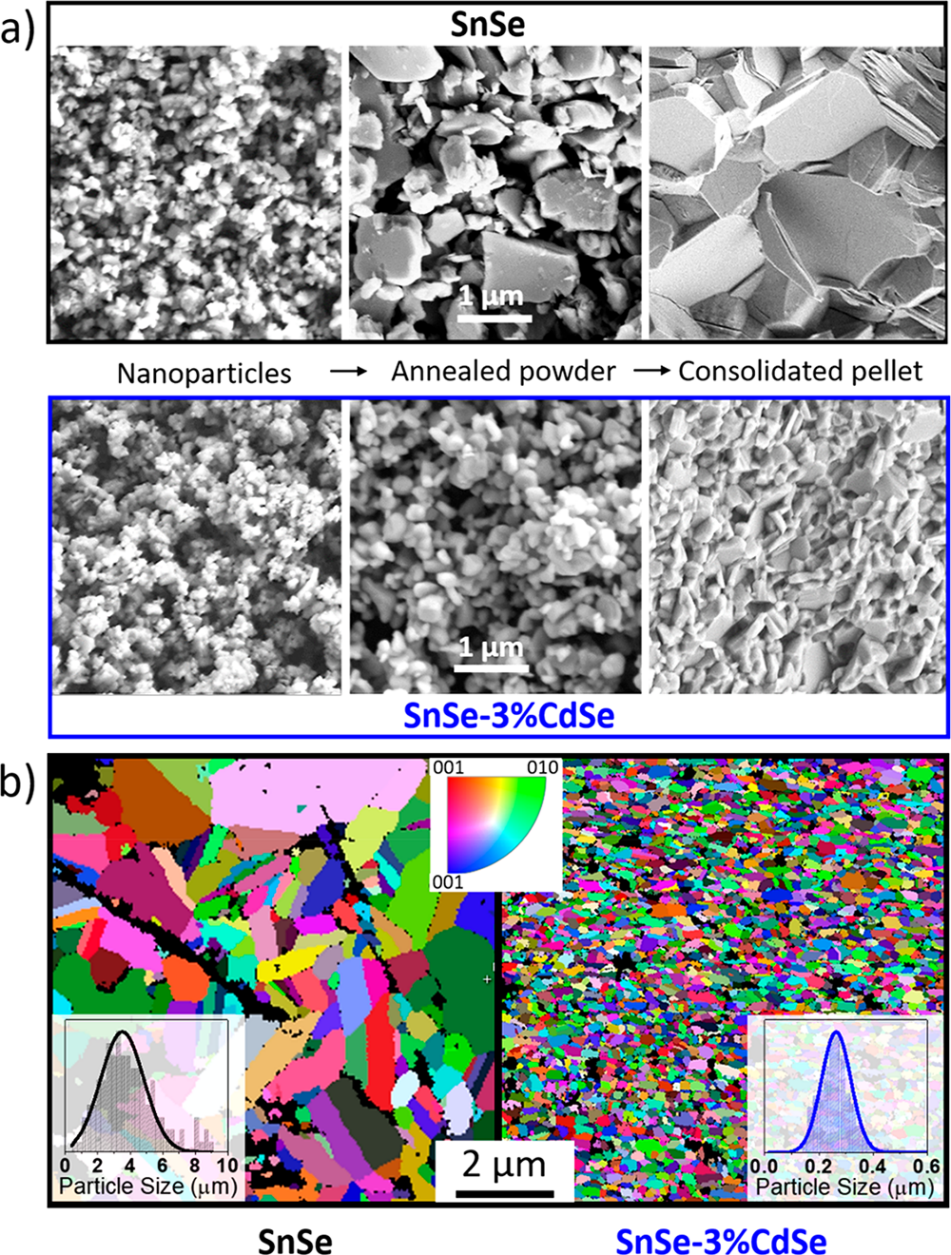
(a) SEM images of SnSe particles, the corresponding annealed powder, and the consolidated pellet without (top, black) and with (bottom, blue) the CdSe-surface treatment. (b) EBSD inverse pole figure maps for bare SnSe (left) and SnSe-3%CdSe (right) pellets; inset images correspond to the particle size histograms.

To understand the mechanism that inhibits grain growth in the presence of CdSe, we analyzed the bare SnSe and SnSe-3%CdSe samples after each processing step (surface treatment, annealing, and consolidation, Figure 3). SEM images indicate that the particle morphology is merely affected by the CdSe surface treatment. Differences appear between the CdSe treated and the untreated samples upon annealing. Despite the fact that both materials show grain growth, the average grain size of bare SnSe increases from 150 ± 50 nm to 680 ± 400 nm, which is 2.5 times larger than that in the presence of CdSe (Figures S4 and S5). The difference in grain growth is more evident after the pressure-assisted sintering step through spark plasma sintering (SPS, 45 MPa, 500 °C). In the presence of CdSe, grain growth is hindered, resulting in relatively smaller crystal domains (Figures 3a and S6) and thus in a higher volume fraction of grain boundaries for the SnSe–CdSe nanocomposites compared to bare SnSe. SnSe samples have an average grain size of ca. 3.5 ± 3.0 μm while SnSe-3%CdSe nanocomposites display grain sizes of ca. 0.3 ± 0.1 μm as shown by electron backscattering diffraction (EBSD) inverse pole figure maps (Figures 3b, S5, and S7).

The sintering process of surface-treated SnSe particles involves the crystallization of CdSe complexes into CdSe NPs.^38^ Control experiments revealed that the complete crystallization of CdSe complexes occurs at 350 °C, yielding ca. 4 nm CdSe NPs (determined by XRD data, Figure S8). At this same temperature, untreated SnSe particles hardly show any difference in grain size compared to the as-synthesized SnSe particles (Figure S9). Considering that the CdSe crystallization temperature is unaffected by the presence of SnSe particles, we conclude that at 350 °C, there is a homogeneous distribution of CdSe NPs at the surface of SnSe particles (Figure S10).^43^ On the basis of the CdSe and SnSe average particle size, CdSe NPs cover approximately 68% of the total surface of SnSe for the SnSe-3%CdSe. Therefore, the corresponding powder densification and grain growth are strongly affected by the changes in atomic diffusion due to the presence of secondary phase CdSe NPs (Figure 3).

In the classical theory of grain growth, the average grain growth rate is proportional to the average rate of grain boundary movement.^44^ However, when second-phase particles are present at the grain boundaries, the driving force for their migrations is reduced, hindering grain growth.^45^ This is known as the Zener pinning effect.^46^ Zener pinning occurs when a grain boundary encounters a second-phase particle, as the particle exerts a drag force on the grain boundary.^47^ In such a situation, the growing grain is subject to two opposing pressures: the driving pressure for growth and the Zener pinning pressure arising from the particles. For a grain to grow further, the net driving pressure should be positive. By adjusting the volume fraction of second-phase particles and their mean radius is possible to kinetically control the final grain size.^48^

The effect of CdSe NPs in the microstructure of SnSe–CdSe nanocomposites is in accordance with grain growth stagnation predicted by the Zener pinning effect. In Figure 2, one can see that as we increase the content of CdSe molecular solute, the average grain size is reduced. Moreover, considering that bare SnSe and all SnSe–CdSe pellets have the same density despite their difference in grain size, we believe that the presence of CdSe NPs favors atomic diffusion along the grain boundaries, i.e., the densification rate. In contrast, atomic diffusion across the grain boundaries is strongly hampered, limiting grain growth. The result is a material with the same density but much smaller grain domains than bare SnSe.^44^

One key point to achieving grain growth inhibition through such a surface treatment is the proper selection of the metal chalcogenide complex. During the thermal processing, the material selected should form NPs at the SnSe particle surface instead of diffusing into its crystal structure creating a solid solution. To satisfy this condition is necessary to choose a material that possesses a positive enthalpy of mixing with a miscibility gap over the processing temperature range. In this case, the enthalpy is a driving force for segregation that prevents the formation of a solid solution.^44^ As observed in their phase diagram,^49^ CdSe and SnSe are immiscible in the whole range of processing temperatures (Figure S13). XRD data corroborates this fact, as there are no changes in the lattice parameters between bare SnSe and SnSe–CdSe nanocomposites (Figures S14 and S15).

**Figure 4:**
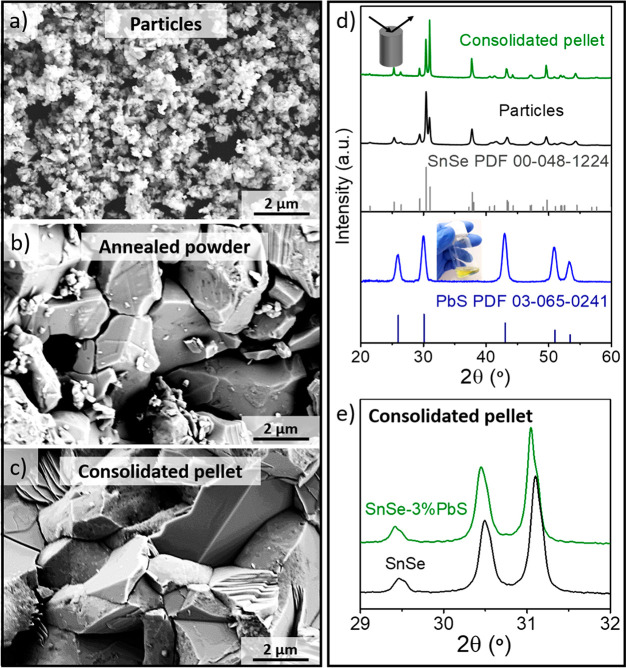
Representative SEM images of (a) PbS-surface-treated SnSe particles, (b) the corresponding annealed powder, and (c) consolidated pellet. (d) XRD diffraction patterns of recrystallized PbS molecular complexes (blue), the surface-modified particles (black), and consolidated pellet in the perpendicular direction to the pressing axis (green). The inset picture corresponds to the PbS molecular solution. The pattern in gray corresponds to the SnSe reference pattern PDF no. 00–048–1224 (JCDPS) and in dark blue to the PbS reference pattern PDF no. 03–065–0241 (JCDPS). (e) Magnification of the XRD pattern for the consolidated pellets with (green) and without (black) PbS surface treatment showing the peak shift due to partial alloying of PbS.

To prove this idea, we chose a different molecular complex with no miscibility gap (PbS) to treat SnSe particles. PbS is known to form a stable solid solution with SnSe up to concentrations of 20%.^50^ When comparing the two composite materials at different processing stages, we observe that in the presence of PbS the grain coarsening is enhanced significantly already in the annealing step and yields pellets with even larger grains than bare SnSe (Figure 4). We associate this with the fact that atomic diffusion across the grain boundaries is promoted during the solid-solution formation, enhancing grain growth. XRD analysis of the SnSe-3%PbS pellet corroborates the solid-solution formation as the lattice parameter changes, from *a* = 11.494 Å to *a* = 11.515 Å (Figures 4e and S16).

### Electronic Transport Properties

**Figure 5:**
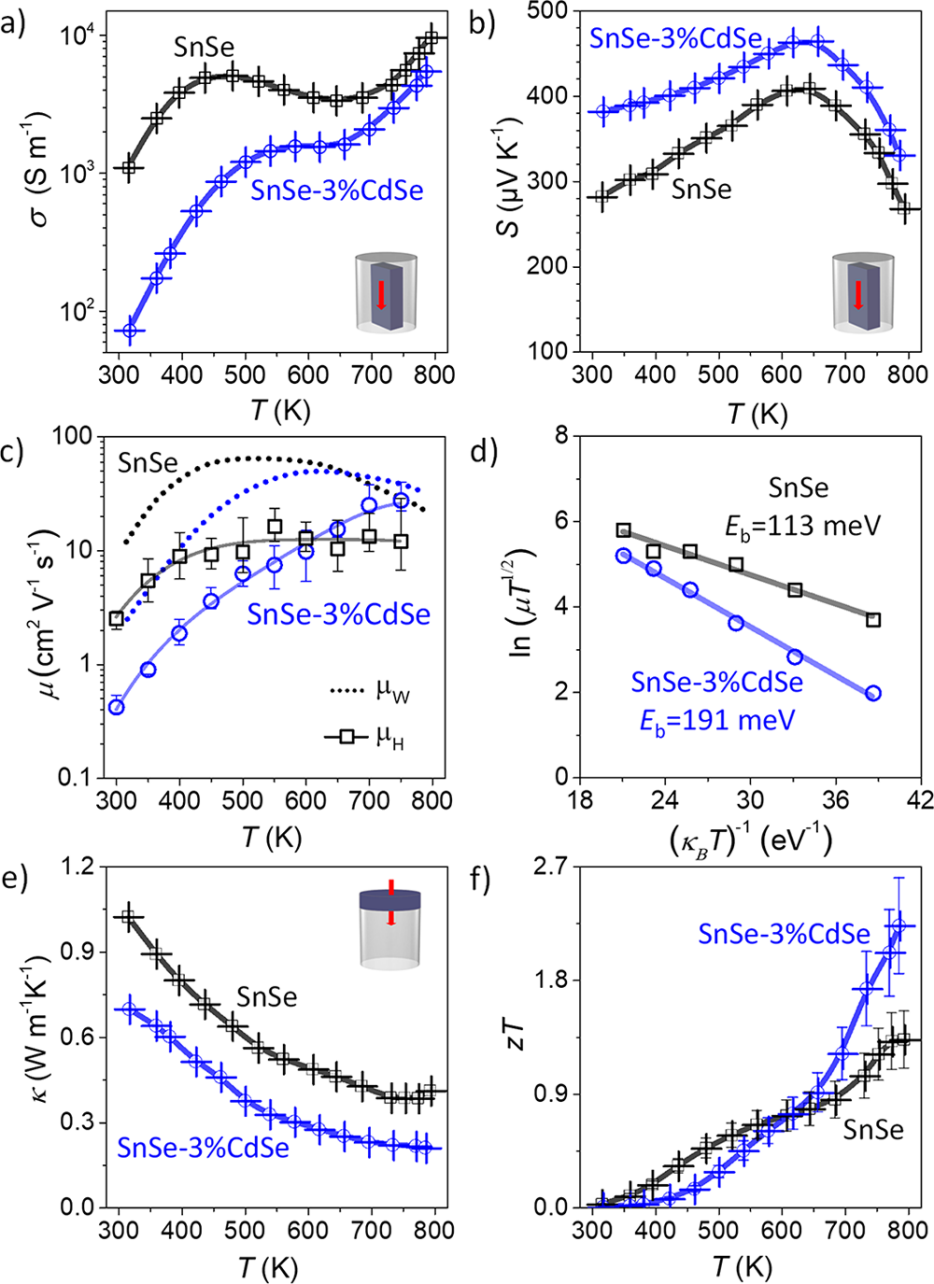
(a) Electrical conductivity, σ. (b) Seebeck coefficient, *S*. (c) Hall and weighted mobilities, μ_H_ and μ_w_, respectively. (d) Fitting for the energy barrier, *E*_b_, (μ ∝ *T*^–1/2^ exp(−*E*_b_/*k*_B_*T*)). (e) Thermal conductivity, κ. (f) Figure of merit, *zT*, for bare SnSe nanomaterial (black) and SnSe-3%CdSe nanocomposite (blue).

The electrical conductivity (σ), Seebeck coefficient (*S*), thermal conductivity (κ), and calculated figure of merit (z*T*) of bare SnSe and SnSe–CdSe nanocomposites with different content of CdSe were measured in the direction parallel (Figure S17) and perpendicular (Figure S18) to the pressure axis. Because materials’ *zT*s in the parallel direction are larger than those in the normal one, we discuss here transport properties in the parallel direction. Also, for clarity, the discussion focuses on comparing bare SnSe and the best performing SnSe–CdSe nanocomposite, which corresponds to 3 mol %, referred to as SnSe-3%CdSe (Figure 5). The data corresponding to other SnSe-*x*%CdSe samples (*x* = 1, 2, and 4) can be found in Figures S17 and S18.

Bare SnSe shows a higher electrical conductivity and a lower positive Seebeck coefficient than those of SnSe-3%CdSe in the whole temperature range (Figures 5a and b). The Hall carrier concentration (*p*_H_) of SnSe-3%CdSe is lower (ca. *p*_H_ = 9 × 10^18^ cm^–3^) than the one obtained for bare SnSe (ca. *p*_H_ = 2 × 10^19^ cm^–3^). CdSe is generally an *n*-type semiconductor,^51^ and it has a much lower electron affinity than that of *p*-type SnSe. Thus, within the SnSe matrix, CdSe injects free electrons to SnSe, inducing band bending in the regions close to CdSe domains. Owing to the small size of the CdSe crystal domains, its Fermi level becomes pinned near the valence band edge, thus trapping holes from the SnSe matrix. Thus, the hole carrier concentration is reduced in the presence of CdSe, which directly translates into an increase of the Seebeck coefficient. Moreover, the presence of larger energy barriers at the interface between CdSe and SnSe allows for even higher Seebeck coefficients due to the filtering of high energy carriers (Figure S19).^52^

The temperature dependence of the electrical conductivity and the Seebeck coefficient of both materials are similar to those previously reported polycrystalline SnSe.^20,31,32,53^ To understand the underlying transport mechanism, we compare the hall mobilities with the calculated weighted mobilities (μ_W_, Figure 5c) according to the equation^54^
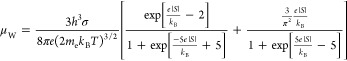
where *h*, *k*_B_, *e*, and *m*_e_, are the Planck’s constant, Boltzmann’s constant, electron charge, and electrons mass. Both mobilities indicate the same behavior.

Bare SnSe and SnSe-3%CdSe nanocomposite show thermally activated conductivity from room temperature up to ca. 500–600 K. In this temperature range, the increase of mobility with temperature reflects the presence of potential barriers due to charge accumulation at the grain boundaries.^55,56^ The energy barrier height (*E*_b_; μ ∝ *T*^–1/2^ exp(*−E*_b_/*k*_B_*T*)) for SnSe–CdSe nanocomposites is 190 meV, while for SnSe, it is 113 meV (Figure 5d). The larger energy barrier of the SnSe-3%CdSe material results in lower mobility at room temperature.^56,57^ At high temperatures, the difference in mobility between both materials is practically negligible. With increasing temperature, the thermally excited carriers reduce the effect of the potential barriers on mobility, and the dominant scattering mechanism in both materials is acoustic phonon scattering.

The Seebeck coefficient in both materials peaks at ca. 650 K, indicating the onset for bipolar conduction. As the material transitions from the *Pnma* to the *Cmcm* phase, the changes in the local bonding translate into differences in the electronic structure, including a reduction of the bandgap from 0.61 to 0.39 eV that favors the thermal excitation of minority carriers.^13,31^ Above 800 K, the material is fully converted to *Cmcm* (Figures S14 and S15) and both the Seebeck coefficient and electrical conductivity stabilize (Figure S20).

### Thermal Transport Properties

The thermal conductivities of both nanomaterials follow the same trend in the whole temperature range. From room temperature up to ca. 790 K, the values decrease monotonically. At higher temperatures, due to the SnSe phase transition to the higher symmetry *Cmcm* phase, the thermal conductivity increases (Figure S20). The temperature at which the *Pnma* fully converts into the *Cmcm* phase was analyzed by differential scanning calorimeter (Figure S21b) and temperature-dependent XRD measurements (Figure S15). Both analyses indicate a complete phase transition at 800 K.

In the whole temperature range, the κ values of SnSe-3%CdSe nanocomposites are almost 50% lower than those of bare SnSe (Figure S22). At the temperature where the thermal diffusivity is minimum (786 K), the *C*_*p*_ value measured in the SnSe-3%CdSe nanocomposite is 0.263 J g^–1^ K^–1^ (Figure S21b) leading to a thermal conductivity of 0.20 W m^–1^ K^–1^ (κ_*lattice*_*=* 0.14 W m^–1^ K^–1^). This value is similar to the lowest reported values for polycrystalline SnSe (Figure S23b).^16,25,32^

**Figure 6:**
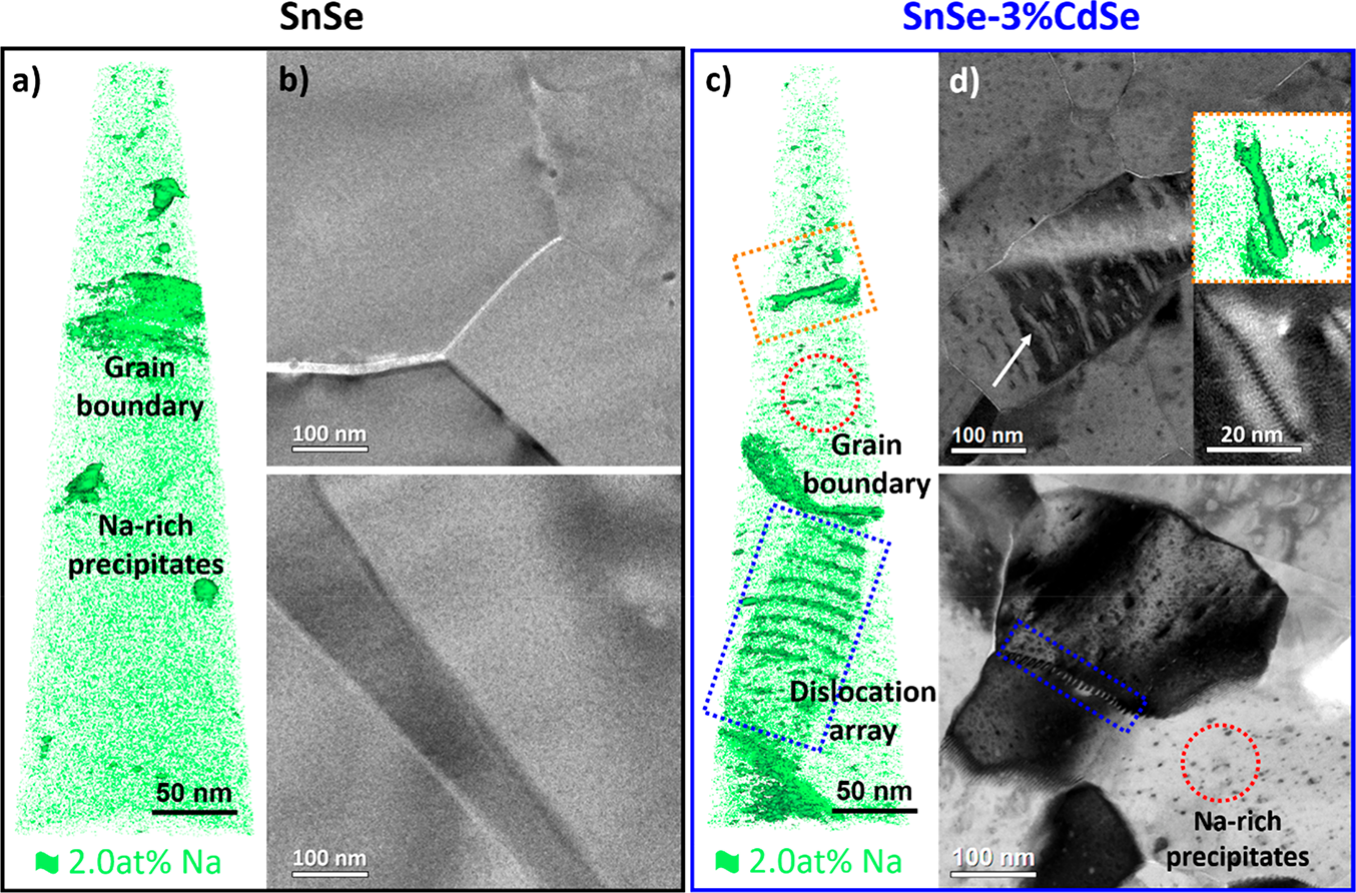
(a) APT characterization showing the 3D distribution of Na and (b) TEM images of different grain boundaries in bare SnSe. (c) APT characterization showing the 3D distribution of Na and (d) TEM images of SnSe-3%CdSe nanocomposites. The correspondence between defects in APT and TEM is highlighted with red, blue, and orange dashed boxes.

To comprehend the origin of such low values, bare SnSe and SnSe–CdSe nanocomposites were further investigated by transmission electron microscopy (TEM) and atom probe tomography (APT, Figures 6).^58,59^

**Figure 7:**
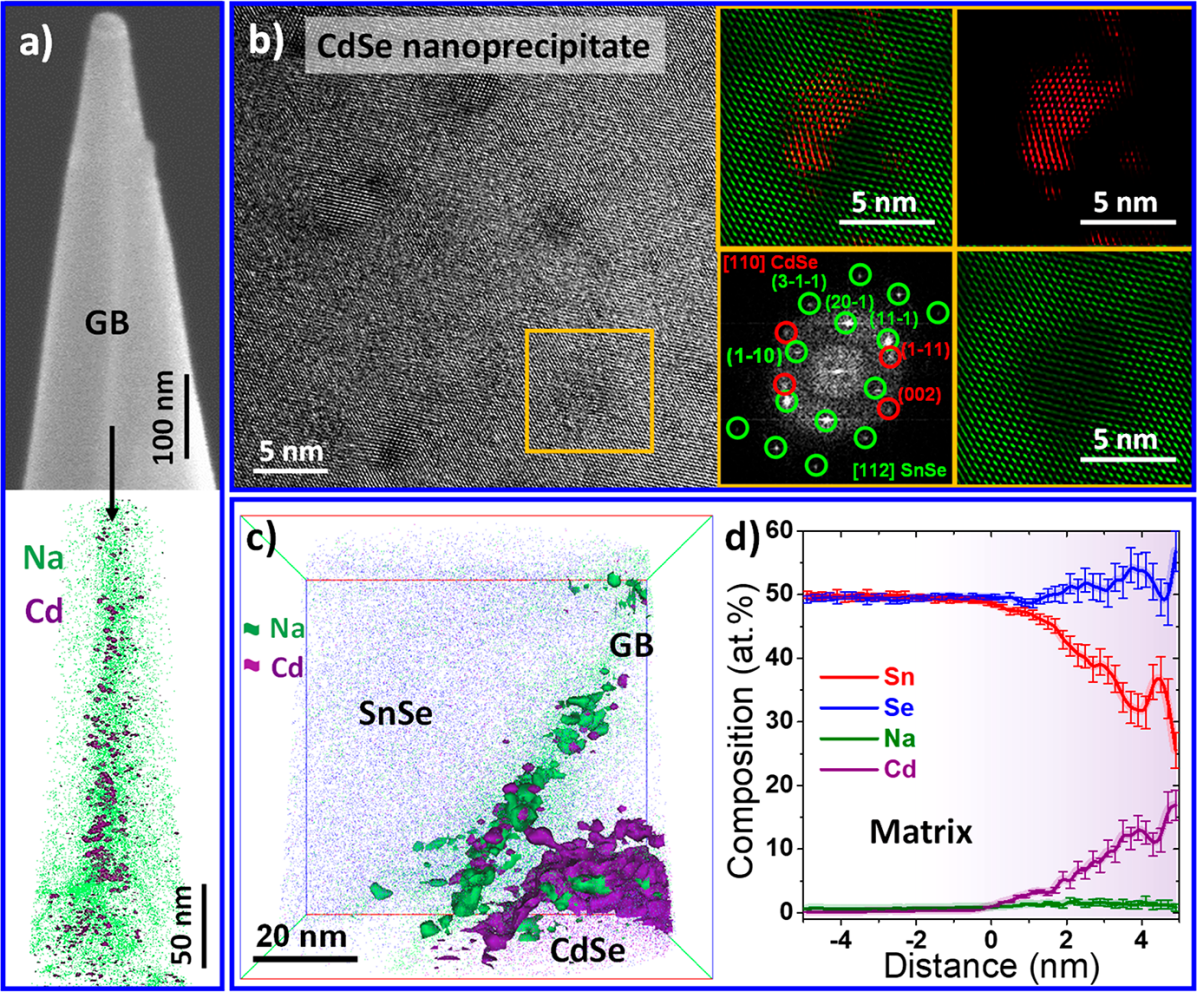
(a) SEM image of the tip (top) used for the APT elemental analysis (bottom) showing the enhanced presence of Na and Cd at the grain boundary (GB). (b) CdSe NP with *Fm*3*m* cubic phase with the corresponding indexed power spectrum from the region marked in yellow and the phase filtered images highlighting the CdSe NP (red) and the SnSe matrix (green). (c) 3D distribution of elements from APT analysis in a region containing a grain boundary and a CdSe NP; (d) composition profile across a CdSe NP.

Na is an unavoidable impurity, yet beneficial for doping, due to the nature of the aqueous-based synthesis and the use of Na salts in the reaction. Figures 6a,c show the isocomposition surface of 2.0 at% Na in green, allowing identifying the 3D distribution of Na in the pellets. In both materials, bare SnSe and SnSe-3%CdSe, Na is found within the grains, at dislocations and grain boundaries, and in Na-rich precipitates (Figures 6a,c and 7). Elemental analysis by inductively coupled plasma optical emission spectroscopy (ICP-OES) and energy-dispersive X-ray spectrometry (EDS) indicate that both pellets contain the same amount of Na (Table S2), yet the defect concentration of defects associate with Na differ in their concentration. The comparison between low-magnification TEM images (Figure 6b,d) and APT data clearly reveals the different microstructure of both materials. SnSe-3%CdSe nanocomposites presented a larger density of grain boundaries, dislocations, planar defects, and Na-rich precipitates (Figures 6d and S24).

Figure 7 shows the analysis carried out with APT and HRTEM for the sample SnSe-3%CdSe. Both techniques revealed the presence of CdSe NPs in the range between 1 and 20 nm (Figures 7a–c and S24). In particular, thanks to power spectrum analysis, we identify that CdSe NP possesses a *Fm*3*m* cubic structure (Figure 7b). Finally, APT uncovered the presence of Cd at the grain boundaries (Figures 7a,d) together with the segregation of Na and depletion of Sn. Due to the variation of chemical composition and likely the structure at the grain boundary, the most accurate way to define the interfacial material at the grain boundary would be grain boundary complexions,^60^ which can generally scatter phonons more strongly than a pure grain boundary.^61^ Such grain boundary complexions are also associated with the enhanced energy barrier for holes that reduces carrier mobility and enhances the Seebeck coefficient through energy filtering effects seen in the charge transport analysis.

The dominant phonon modes in SnSe have mean free paths of a similar length to the defects found in SnSe-3%CdSe nanocomposite, such as atomic impurities Na and Cd, CdSe NPs and Na-rich precipitates ranging from 1 to 20 nm, and grain boundary complexions.^62^ This explains the significant reduction in the overall thermal conductivity with respect to bare SnSe, where the density of such defects is significantly lower. Moreover, the surface treatment with CdSe molecular solution may reduce the overall content of oxide species in the final composite, therefore, having also a positive effect on the thermal conductivity.^16,17^ Such reduction of the thermal conductivity leads to a state-of-the-art figure of merit in solution-processed SnSe of 2.2 at 786 K, comparable to the best performing polycrystalline SnSe (Figure S23a). Additionally, the results obtained from SnSe-3%CdSe pellets exhibit excellent stability (Figure S26) and repeatability from sample to sample (Figure S27).

## Conclusions

In summary, we presented a scalable, simple, and economical method to produce high-performance polycrystalline SnSe thermoelectric materials. Specifically, we optimized a water-based synthesis protocol to obtain large quantities of SnSe particles and developed a surface treatment to (i) inhibit grain growth during consolidation and operation, (ii) introduce CdSe NPs within the range 1–20 nm, (iii) create grain boundary complexions, and (iv) enhance the number of defects at multiple length scales, such as atomic impurities, planar defects, dislocations, and Na-rich precipitates. The presence of scattering sources at all relevant length scales improved the figure of merit from *zT* = 1.3 in bare SnSe to *zT* = 2.2 in SnSe–CdSe nanocomposites. We believe that the strategy presented here for inhibiting grain growth is of great significance beyond the thermoelectric field because it tackles grain growth in semiconductor nanocomposites.

## Methods

### Chemicals

Tin(II) chloride dihydrate (SnCl_2_·2H_2_O, 98%), sodium hydroxide (NaOH, pellets 98%), sodium borohydride (NaBH_4_, 98%), cadmium oxide (CdO, 99.99%), lead(II) oxide (PbO, 99.99%), and *N*-methylformamide (MFA, 99%) were purchased from Fisher Scientific. Selenium powder (Se, 100 mesh, ≥99.5%), ethylenediamine (en, 99%), 1, 2-ethanedithiol (EDT, ≥95.0%), anhydrous acetone (extra dry), and ethanol (95%) were purchased from Sigma-Aldrich. All chemicals were used as received without further purification. Syntheses were carried out using a vacuum/dry argon Schlenk line.

### SnSe Particle Synthesis

SnSe particles were prepared following previously reported by Gregory et al.,^37^ with slight modifications. In a typical synthesis, NaBH_4_ (6.053 g, 160 mmol) was first dissolved in 400 mL of deionized water, and then Se powder (6.314 g, 80 mmol) was slowly added into the solution. Stirring should be avoided during this step due to the strong evolution of hydrogen gas. Once the bubbling finished, stirring was resumed under Ar flow until the solution became transparent indicating the complete reduction of Se. In parallel, NaOH (30 g, 750 mmol) and SnCl_2_·2H_2_O (16.247 g, 72 mmol) were mixed with 360 mL of deionized water. The mixture was stirred at room temperature under Ar flow until complete dissolution. At this point, the solution was heated under reflux to its boiling point (ca. 101.3 °C). The freshly prepared Se solution was rapidly injected into the boiling Sn (II) solution, and the temperature dropped to ∼70 °C. Upon injection, the reaction mixture turned black indicating the particle formation. The temperature was allowed to recover to ca. 101.3 °C, and this temperature was maintained for 2 h. To purify the as-synthesized particles, the mixture was decanted, and the transparent supernatant solution was carefully discarded. The remaining crude solution (ca. 120 mL) was purified by 3 precipitation/redispersion cycles with deionized water and ethanol alternatively. In the first cycle, 120 mL of deionized water were added into the crude solution, and the particles were separated by centrifugation (6000 rpm, 5000 *g*, 1 min). Then, the particles were redispersed in 105 mL of ethanol and centrifuged (8000 rpm, 5 min). In the second cycle, 120 mL of deionized water was added to solubilize the remaining impurities and disperse the particles, which were precipitated (9000 rpm, 5 min). Afterward, 105 mL of ethanol were employed to redisperse and precipitate the particles (8000 rpm, 5 min). These same steps were repeated for a third purification cycle. Washed particles were dried under vacuum overnight at room temperature and kept in the glovebox for further use.

### CdSe Molecular Complexes Preparation

The CdSe molecular complexes (87 mg/mL) were obtained by mixing stoichiometric amounts of CdO (4 mmol) and Se powder (4 mmol) with en (8 mL) and EDT (0.8 mL) in a N_2_-filled vial following a modified approach to the developed by Brutchey et al.^38^ The mixture was agitated for ∼5 min at room temperature until complete dissolution. All the CdSe molecular complexes solutions were prepared fresh before blending with SnSe particles in MFA, due to their limited stability. Similarly, PbS molecular complexes (109 mg/mL) were obtained by mixing 1 mmol of PbO with 2 mL of en and 0.2 mL of EDT in a N_2_-filled vial.

### Particle Surface Treatment

All surface treatments were carried out in an inert atmosphere (N_2_). Different amounts (1%: 0.44 mL, 2%: 0.88 mL, 3%: 1.32 mL, and 4%: 1.76 mL) of CdSe molecular complexes solution were mixed with different amounts of MFA (ca. 50 μL/mL). We denoted these mixtures as CdSe-MFA solutions. Then, these CdSe-MFA solutions were combined with 4.0 g of dried SnSe particles and vigorously stirred at room temperature for 48 h. After that, acetone was added to the mixture and the particles were precipitated by centrifugation. Subsequently, the CdSe molecular complex capped SnSe particles were washed one more time with acetone, centrifuged, and dried under vacuum yielding a fine powder. SnSe particles were treated with 3% PbS molecular complexes following the exact same process as for the SnSe–CdSe system.

### Bulk Nanomaterial Consolidation

Dried SnSe-*x*%CdSe (*x* = 0, 1, 2, 3, and 4) nanocomposites were first annealed at 500 °C for 60 min under a forming gas (95% N_2_ + 5% H_2_) flow inside a tube furnace with the heating rate of around 10 °C/min. Afterward, the annealed nanopowder was ground with an agate mortar and loaded into a graphite die lined with graphite paper inside the glovebox. The nanopowder was then consolidated into cylinders under vacuum (Ø 8.6 mm × *h* = 12 mm) in an AGUS PECS SPS System-Model SPS 210Sx by applying an axial pressure of 45 MPa at 500 °C for 5 min. All consolidated cylinders presented relative densities above 92% of the theoretical value. Finally, all the cylindrical pellets were annealed in forming gas (95% N_2_ + 5% H_2_) static atmosphere for 1 h at 550 °C (ca. 4 °C/min). These cylinders were cut in two normal directions, i.e., parallel to the pressing direction and within the cylinder plane, into discs and rectangular bars (Figure S28).

### Structural and Chemical Characterization

XRD measurements were carried out on a D8 ADVANCE diffractometer (Bruker, Germany) with Cu Kα radiation from 20 to 60° with a resolution of 0.01° and time step of 0.1 s. Temperature-dependent XRD patterns were collected in a reactor chamber with sample spinning and height correction (XRK 900, Anton Paar). The temperature was ramped from 50 to 550 °C at a rate of 50 °C/min and held for 10 min before each measurement. The size and morphology of initial particles, annealed nanopowders, and sintered pellets were examined by field-emission scanning electron microscopy operated at 5.0 kV (FE-SEM Merlin VP Compact, Zeiss). The overall material composition was investigated by using EDS (Octane Elite EDS, Oxford) attached to the SEM operated at 15.0 kV. EBSD studies were used to estimate the average grain size of the consolidated materials. The samples for EBSD were polished with a Neutral Alumina Suspension (OP-AN Struers No. 40700054). The EBSD measurements were carried out on Zeiss FEG SEM Merlin microscope with OXFORD EBSD Symmetry and OXFORD EDS Ultim Max 170 using the software Aztec 4.3HF1 and a microscope at an accelerating voltage of 200 kV with a JEOL silicon drift detector (SDD). Scanning transmission electron microscope (STEM) characterization of the CdSe surface-coated SnSe samples has been carried out using a JEOL JEM2800 microscope operated at 200 kV with a point to point resolution of 0.14 nm. HRTEM was also done with (TECNAI F20, FEI) microscope operated at 200 kV with a point to point resolution of 0.14 nm. TEM specimens were prepared by Focused Ion Beam (FIB) (Helios Dual Beam Nanolab, FEI). In order to evaluate the crystal structure, 3D atomic models of the matrix and the precipitates were created using the Rhodius software platform.^63,64^ These models were used for the HRTEM image simulation with the STEM_CELL software.^65,66^ The models used for the simulation of the image were those created by Rhodius. Needle-shaped APT specimens were prepared by a standard “lift-out” method in an SEM/FIB dual-beam focused ion beam microscope (Helios NanoLab 650, FEI). APT measurements were carried out on a local electrode atom probe (LEAP 4000X Si, Cameca) by applying 10 ps, 5 pJ ultraviolet laser pulses (355 nm). The pulse repetition rate was 200 kHz, and the detection rate was set as 1 ion per 100 pulses (1%) on average. The measurement base temperature of specimen was 30 K to minimize surface migration. The detection efficiency was 50% owing to the open area of the microchannel plates, and the ion flight path was 160 mm. APT data was processed with the software package IVAS 3.8.0. The overall material composition was investigated by using an Oxford EDS apparatus attached to the Zeiss Auriga SEM at 15.0 kV and by optical emission spectroscopy by means of inductively coupled plasma (ICP) on the ICPE-9820 system.

### Thermoelectric Property Measurement

Seebeck coefficients were measured by using a static direct current (DC) method. Electrical resistivities were obtained by a standard four-probe method. Both Seebeck coefficient and electrical resistivity were simultaneously measured in a Linseiss LSR-3 system from room temperature to ca. 790 K, under helium atmosphere. Samples were held between two alumel electrodes and two probe thermocouples with spring-loaded pressure contacts. A resistive heater on the lower electrode created temperature differentials in the sample to determine the Seebeck coefficient. We estimate an error of ca. 4% in the measurement of both electrical conductivity and Seebeck coefficient. Combining the uncertainties of electrical conductivity and Seebeck coefficient, the uncertainty of the power factor is ca. 12%. The results presented here are an average of the results obtained after measuring 3 pellets produced under identical conditions; measurements between different samples have standard deviations below 10%. Additionally, each pellet was measured 3 times; a difference in the temperature dependent thermoelectric properties is only found between the first up and down measurements (Figure S26). In subsequent measurements, the values remain stable. To avoid this hysteresis, before transport measurements the samples undergo a temperature treatment detailed in “Bulk Nanomaterial Consolidation” section. A Xenon Flash Apparatus (XFA 500, Linseis) and a Laser Flash Analyzer (LFA 1000, Linseis) were used to determine thermal diffusivity of the samples with an estimated error of ca. 5%. The total thermal conductivity was calculated by κ = λ*C*_*p*_ρ, where λ is the thermal diffusivity, *C*_*p*_ is the heat capacity, and ρ is the mass density. Temperature-dependent *C*_*p*_ values were also evaluated by DSC (DSC 404 F3, Netzsch) and calculated by the *C*_*p*_ ratio method with a sapphire standard using the Proteus software (Netzsch). Measurements were carried out under high purity N_2_ flow in a Pt–Rh/Al_2_O_3_ crucible. The sample was first preheated to 50 °C and keep for 10 min to avoid a heat hook and then heated to 550 °C with a rate of 10 °C/min and keep for 10 min, before cooling inside the instrument. *C*_*p*_ was also estimated from the Dulong–Petit limit (3R law). The densities (ρ) were measured using the Archimedes’ method with a ca. 2% error. Figure S21d displays a comparison of the *zT* values obtained when considering the experimental *C*_*p*_ or the calculated *C*_*p*_. As a consequence, the combined uncertainty for all measurements involved in *zT* determination shown in the plot is estimated to be ca. 17%. To avoid cluttering the plots, error bars were not included in the figures. The results reported in this work were measured at our lab in IST Austria as well as by Linseis Messgeräte GmbH (Germany). Temperature dependent Hall charge carrier concentrations (*p*_H_) and mobilities (μ_H_) were measured from 300 to 750 K with the Van der Pauw method using a magnetic field of 0.6 T (ezHEMS, NanoMagnetics) (Figure S29a). Values provided correspond to the average of 10 measurements, and the estimated error is ca. 10%.

## Supporting Information Available

Additional characterization data including TEM, HRTEM, SEM, EBSD, XRD, thermoelectric properties, and literature comparison (PDF)
