# Supplementary material for: Defect Engineering in Solution-Processed Polycrystalline SnSe Leads to High Thermoelectric Performance
Source: ACS Nano. 2021 Sep 22;16(1):78–88. doi: 10.1021/acsnano.1c06720 (PMC8793148; doi:10.1021/acsnano.1c06720)
Supplement: Supplementary file 1 — nn1c06720_si_001.pdf [file nn1c06720_si_001.pdf]

SUPPORTING INFORMATION

Defect Engineering in Solution-Processed Polycrystalline SnSe Leads to High Thermoelectric Performance

Yu Liu,[†] Mariano Calcabrini,[†] Yuan Yu,[‡] Seungho Lee,[†] Cheng Chang,[†] Jérémy David,[§] Tanmoy Gosh,[†] Maria Chiara Spadaro,[§] Chenyang Xie,[#] Oana Cojocaru-Mirédin,[‡] Jordi Arbiol,[§,¶] Maria Ibáñez[†,]*

† IST Austria, Am Campus 1, 3400 Klosterneuburg, Austria.

‡ RWTH Aachen, I. Physikalisches Institut (IA), Sommerfeldstraße 14, 52074 Aachen, Germany.

§ Catalan Institute of Nanoscience and Nanotechnology (ICN2), CSIC and BIST, Campus UAB, Bellaterra, 08193 Barcelona, Catalonia, Spain.

Department of Physics, INTE & Barcelona Multiscale Res. Center, Universitat Politècnica de Catalunya, Avda. Eduard Maristany 16, 08930 Barcelona, Catalunya, Spain

¶ ICREA, Pg. Lluis Companys 23, 08010 Barcelona, Catalonia, Spain

* E-mail: M. Ibañez: mibanez@ist.ac.at

Contents

The tracking process of adsorption of CdSe species on the SnSe surface S3
XRD patterns of SnSe and SnSe-x%CdSe nanocomposites S3
SEM images of SnSe-3%CdSe nanocomposites at the different stages S4
SEM images of annealed SnSe-x%CdSe nanopowders S5
Grain size evolution study for bare SnSe and SnSe-3%CdSe S5
SEM images at different magnifications of SnSe and SnSe-3%CdSe pellets S6
EBSD microstructure of SnSe and SnSe-3%CdSe pellets S7
XRD pattern of recrystallized CdSe S7
SEM images of annealed SnSe powder at 350°C S8
EDS elemental mapping for SnSe-3%CdSe S8
Surface treatment S9
Thermogravimetric analyses S10
SnSe-CdSe phase diagram S11
High-temperature XRD analyses of SnSe and SnSe-3%CdSe S12
Lattice parameters and unit cell volume of SnSe-3%PbS pellet S13
TE properties of SnSe-CdSe samples with different content of CdSe S14
Band structure changes in SnSe induced by the CdSe NPs S15
TE properties of SnSe and SnSe-3%CdSe measured in parallel direction S16
Heat capacity C_p of SnSe-3%CdSe S17
Percentage variations in the TE properties of SnSe-x%CdSe compared to SnSe S18
Lattice thermal conductivity (κ_L) calculation S18
Literature comparison S19
TEM images of SnSe-3%CdSe sample S20
Material stability and repeatability S22
Cylindrical pellet cutting S23
Theoretical zT prediction S24
Pellet density and composition S26
References S27

The tracking process of adsorption of CdSe species on the SnSe surface

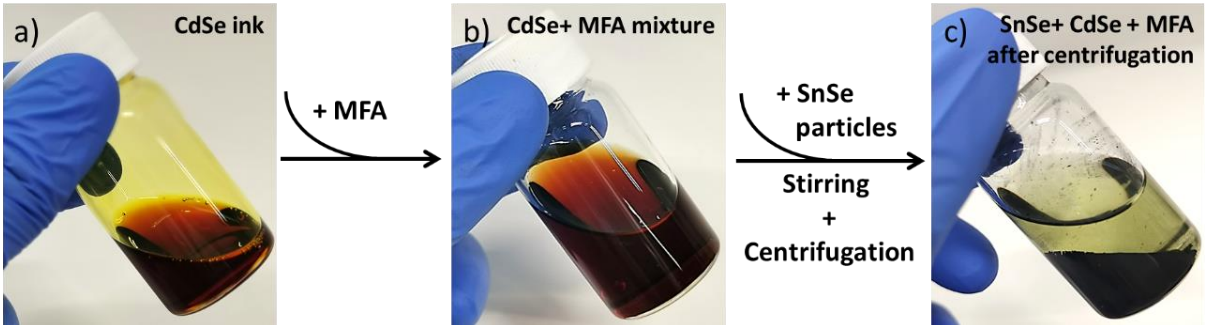

Figure S1. The tracking process of adsorption of CdSe species on the SnSe surface: a) the initial CdSe molecular complexes solution in en-EDT mixture; b) CdSe molecular complexes in N-methyl formamide; c) SnSe particles stirred with CdSe molecular complexes in N-methylformamide for 48 h after centrifugation.

XRD patterns of SnSe and SnSe-x%CdSe nanocomposites

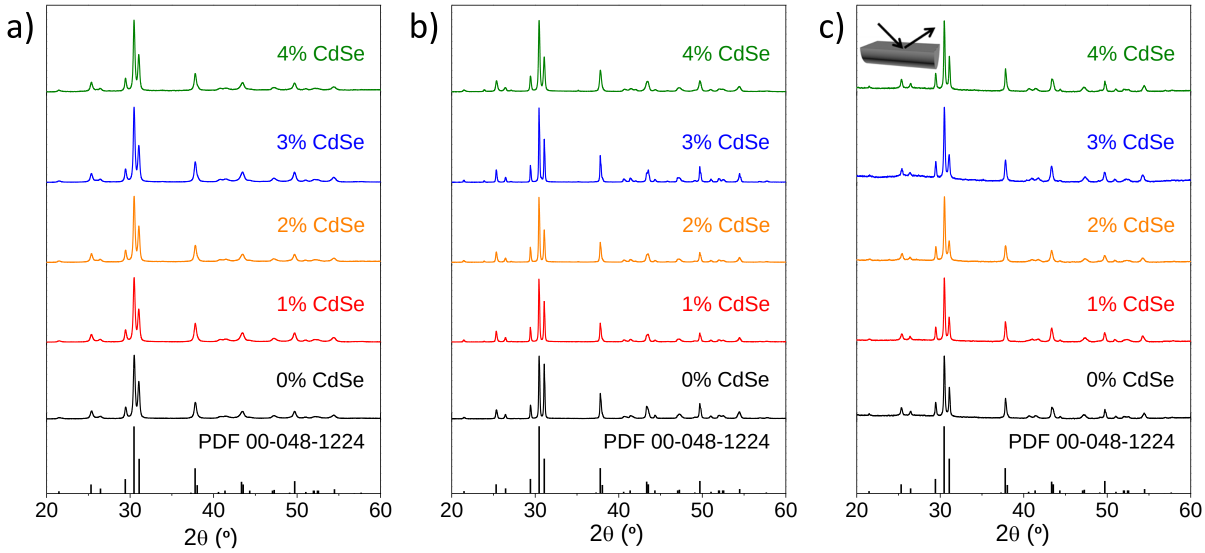

Figure S2. XRD patterns of SnSe particles and SnSe-x%CdSe (x=1, 2, 3, and 4) nanocomposites a) before, b) after annealing and c) the corresponding XRD patterns of these consolidated pellets in the direction parallel to the pressing axis including the SnSe reference pattern PDF 00-048-1224.

SEM images of SnSe-3%CdSe nanocomposites at the different stages

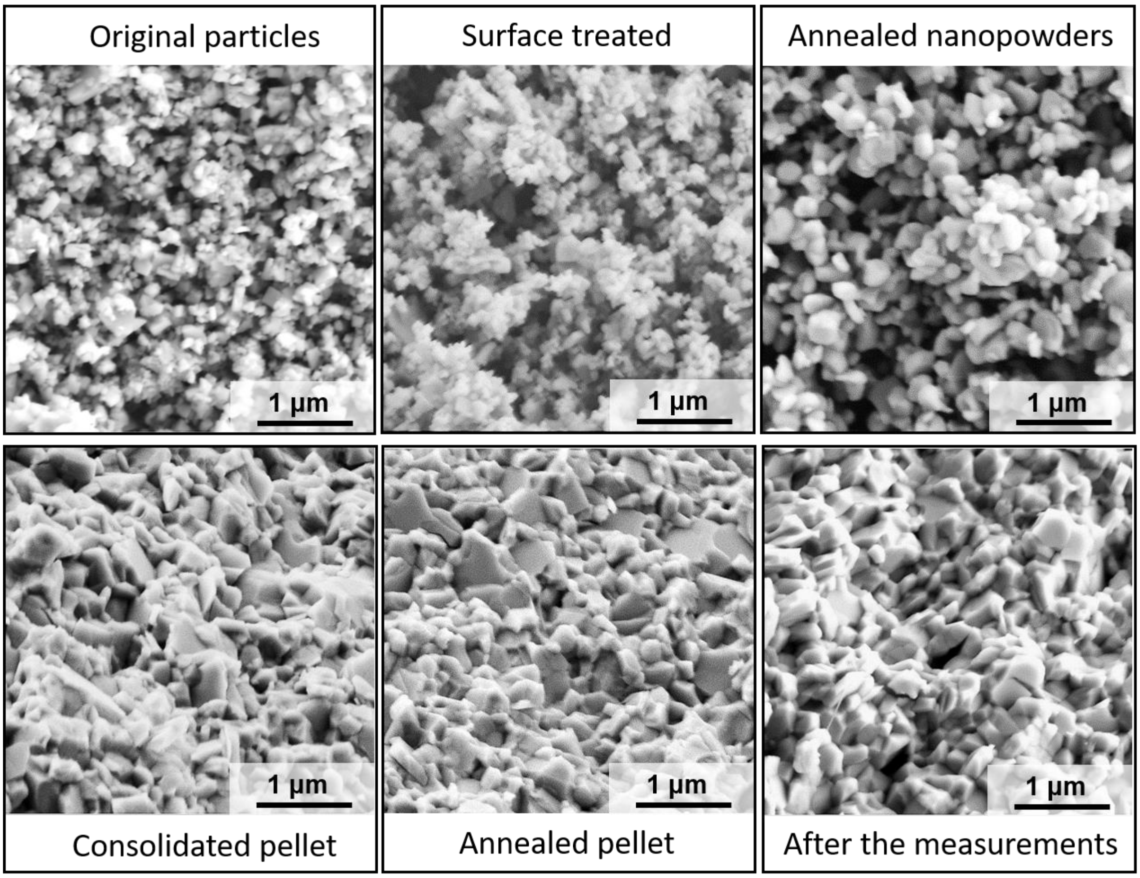

Figure S3. SEM images of the material at the different stages of the process for the SnSe-3%CdSe nanocomposites. In the top row: as-synthesized SnSe particles, surface treated (3%CdSe) particles, and annealed SnSe-3%CdSe nanopowder (500 °C, 60 min, forming gas). In the bottom row, the corresponding consolidated SnSe-3%CdSe pellet (SPS, 500 °C for 5 min at 45 MPa), the annealed SnSe-3%CdSe pellet (823 K, 1 h, forming gas), and the SnSe-3%CdSe pellet after the heating and cooling cycles (transport measurement).

SEM images of annealed SnSe-xCdSe nanopowders

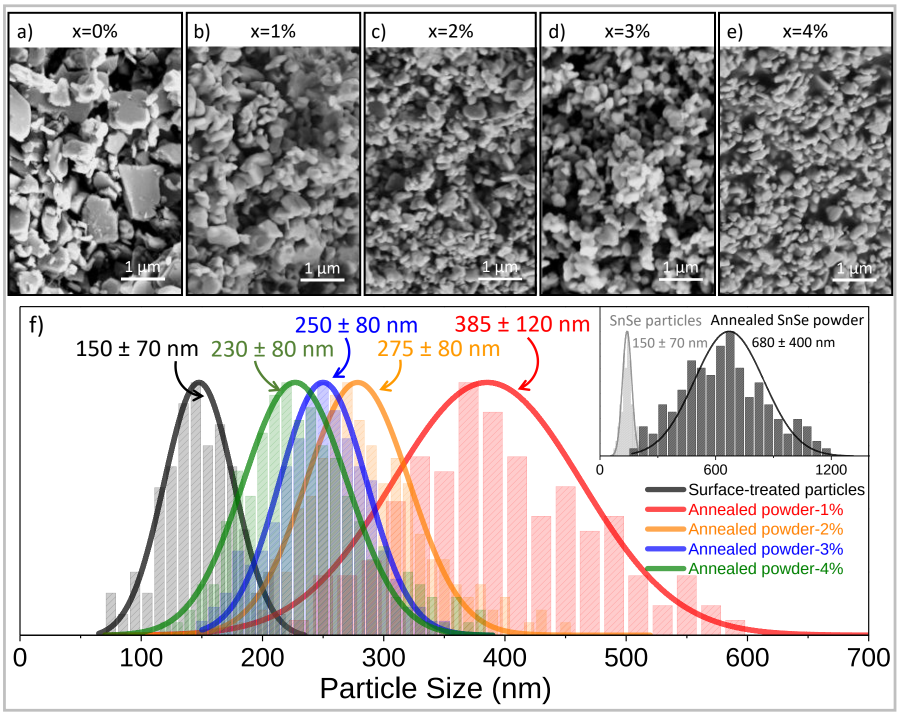

Figure S4. Representative SEM images of annealed nanopowders: a) SnSe, b) SnSe-1%CdSe, c) SnSe-2%CdSe, d) SnSe-3%CdSe and e) SnSe-4%CdSe. f) The particle size distributions histograms and corresponding average size obtain by measuring more than 100 particles.

Grain size evolution study for bare SnSe and SnSe-3%CdSe

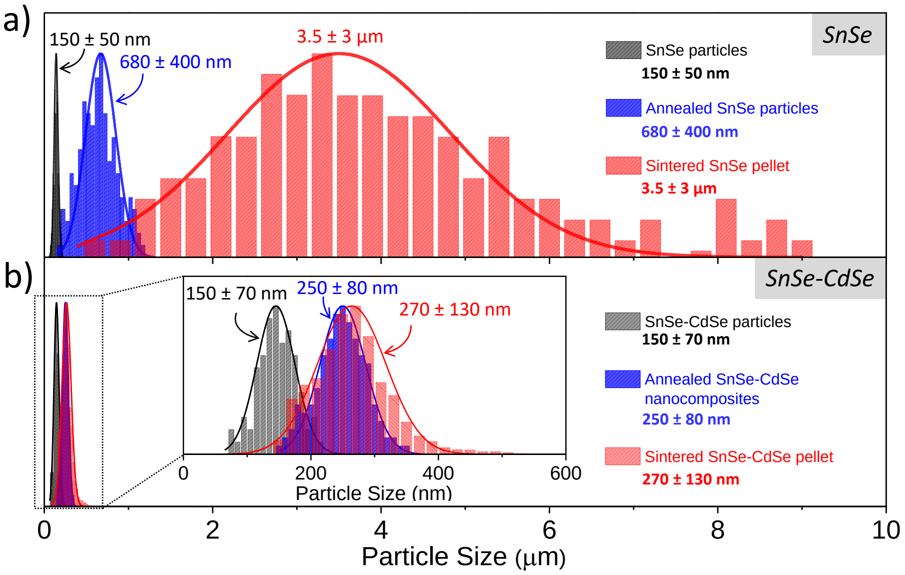

Figure S5. Grain size evolution study for a) bare SnSe and b) 3% CdSe coated SnSe particles. The sizes for the nanopowders were obtained by measuring more than 100 particles in the SEM images; for the pellets, the grain size distributions were obtained from SEM/EBSD.

SEM images at different magnifications of SnSe and SnSe-3%CdSe pellets

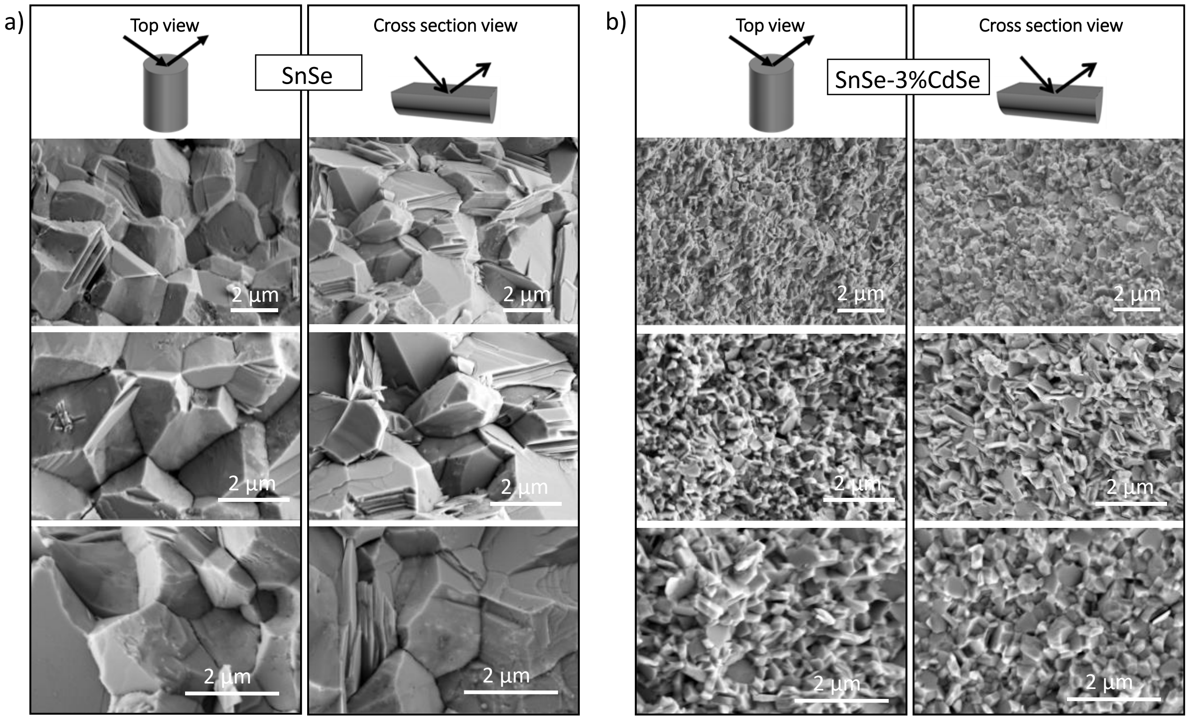

Figure S6. Representative SEM images at different magnifications of the bulk a) SnSe and b) SnSe-3%CdSe nanocomposites in both directions, top-view and cross-section view. All these SEM images were obtained after the pellets were annealed at 823 K for 1h and underwent through several measurement cycles. For details, please refer to the section "*bulk nanomaterial consolidation*" in the main text.

EBSD microstructure of SnSe and SnSe-3%CdSe pellets

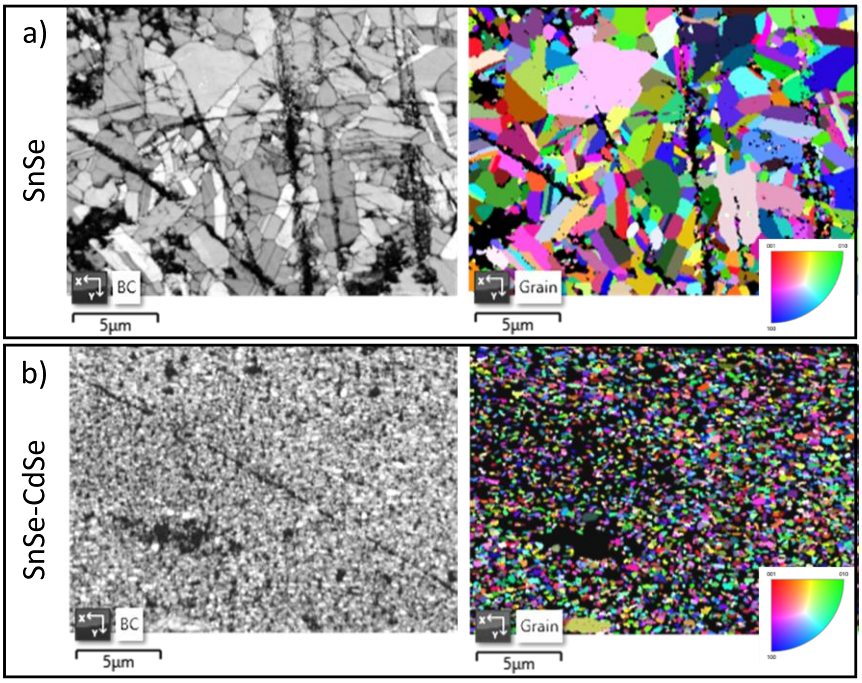

Figure S7. EBSD microstructure of a) bare SnSe and b) SnSe-3%CdSe nanocomposite pellet. On the left of a) and b): the band contrast images of bare SnSe and SnSe-3%CdSe nanocomposite pellet, respectively. On the right of a) and b): the grain color images of bare SnSe and SnSe-3%CdSe nanocomposites, respectively.

XRD pattern of recrystallized CdSe

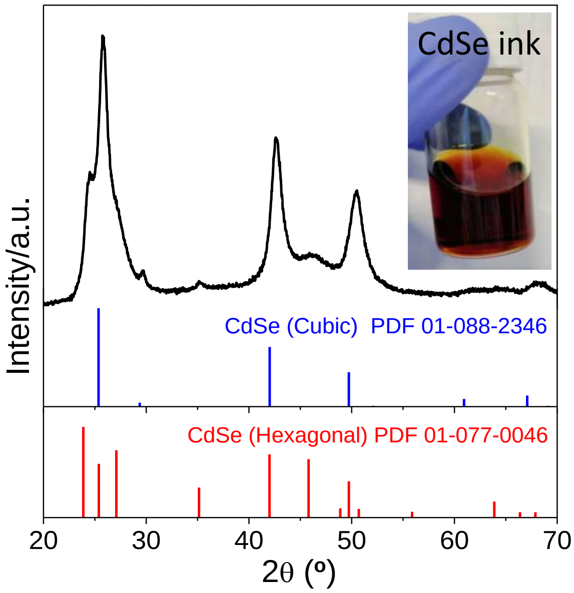

Figure S8. XRD pattern of recrystallized CdSe upon annealing at 350 °C, the inset shows the initial molecular complexes solution of CdSe in the en-EDT mixture including reference

patterns for cubic and hexagonal CdSe. The crystalline CdSe size calculated by the Scherrer equation is approximate 3.1~4.3 nm.

SEM images of annealed SnSe powder at 350°C

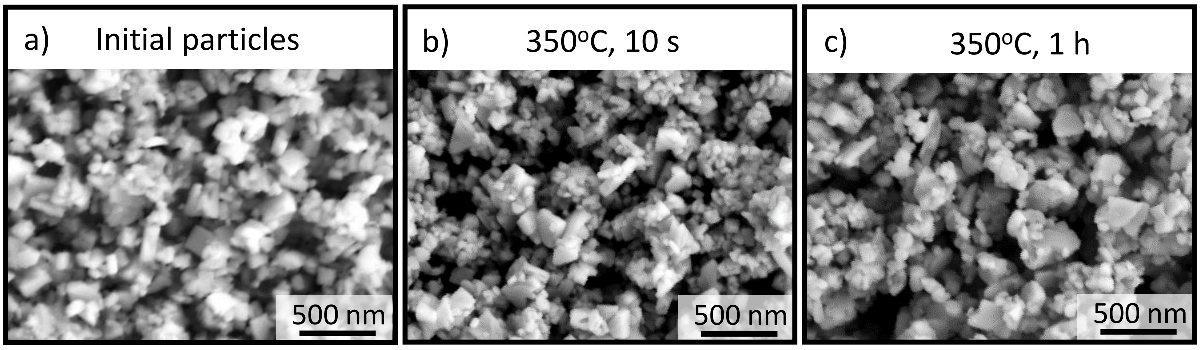

Figure S9. Representative SEM images of a) initial SnSe paticles; b) annealed SnSe nanopowder at 350°C for 10 s; c) annealed SnSe nanopowder at 350°C for 1 h. It can be seen there is only little difference in the grain size of bare SnSe annealed up to 350°C.

EDS elemental mapping for SnSe-3%CdSe

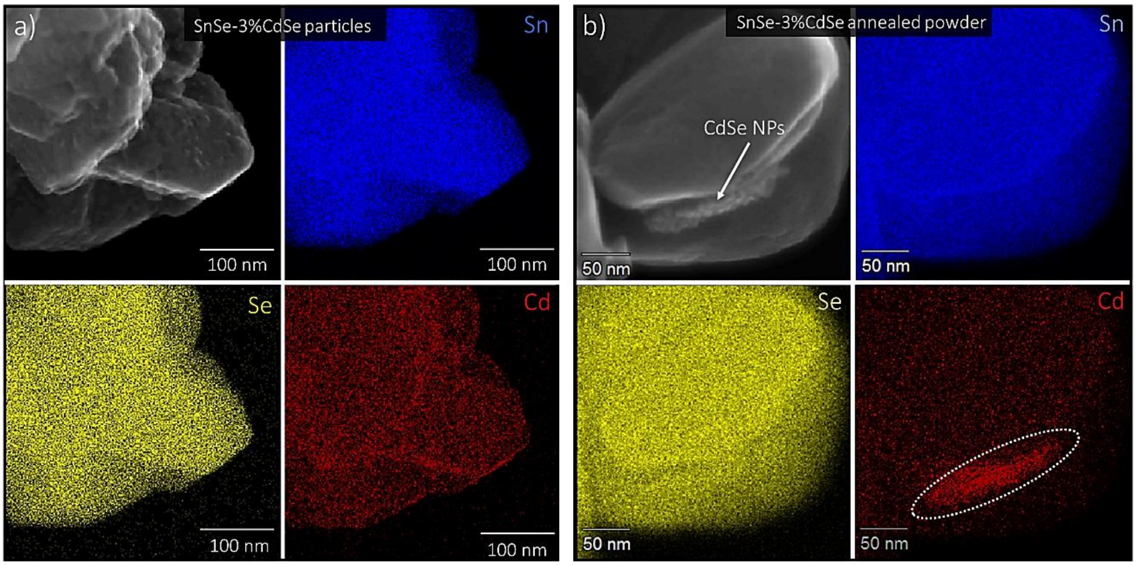

Figure S10. Representative scanning transmission electron microscopy (STEM) micrograph of SnSe-3%CdSe particles before (a) and after (b) annealing with the corresponding STEM-EDS elemental mapping for Sn (blue), Se (yellow), and Cd (red). The contrast of the Cd map has been enhanced to help the reader visualize the presence of Cd. Hence the display intensity cannot be used to quantify the content of Cd.

Surface treatment

To decouple the role of the thiol-amine mixture from the presence of CdSe, we mixed SnSe particles with MFA and then add the thiol-amine mixture without the presence of CdSe and followed the same process to obtain a pellet. SEM images of the consolidated particles treated with and without thiol-amine mixture showed similar grain size. Indeed, the en+EDT mixture can slightly dissolve SnSe; however, as we mix it with MFA, SnSe solubility is highly reduced and one can observe that the particle showed slightly softened edges when treated in MFA containing thiol-amine mixture.

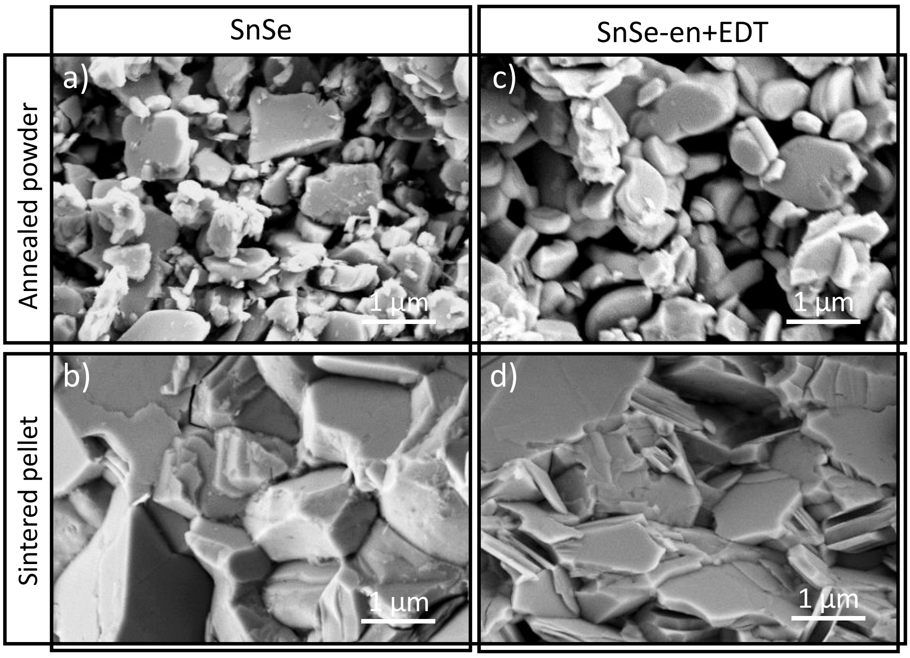

Figure S11. Representative SEM images of the a) annealed SnSe particles, b) corresponding cross-section of sintered SnSe pellet; c) annealed SnSe previously treated with the en+EDT mixture, and d) the corresponding cross-section of the sintered pellet.

Thermogravimetric analyses

The thermogravimetric analyses (TGA) were performed on a LINSEIS, TGA 1000 system on bare SnSe particles, and SnSe-3%CdSe nanocomposites to determine amounts residual of solvents after nanocomposites purification, heating from room temperature to 850°C at a heating rate of 10°C/min under Ar flow.

The SnSe-3%CdSe nanocomposites showed a weight loss of 3.2%, which we associate to: i) the decomposition of the CdSe molecular complexes from (ethylenediamine, boiling point ~116 ºC and 1, 2-Ethanedithiol, boiling point ~146 ºC), and ii) the evaporation of the residual solvent N-Methylformamide (boiling point ~182.6 ºC). Finally, for both the SnSe and SnSe-3% CdSe a weight loss at temperatures above 700 ºC, close to the melting point of SnSe (861 ºC), is observed, corresponding to the removal of lattice chalcogens.

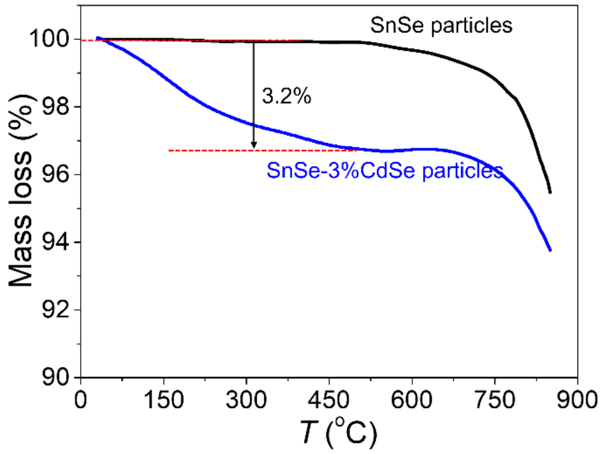

Figure S12. TGA of the original SnSe particles, and SnSe-3%CdSe particles.

SnSe-CdSe phase diagram

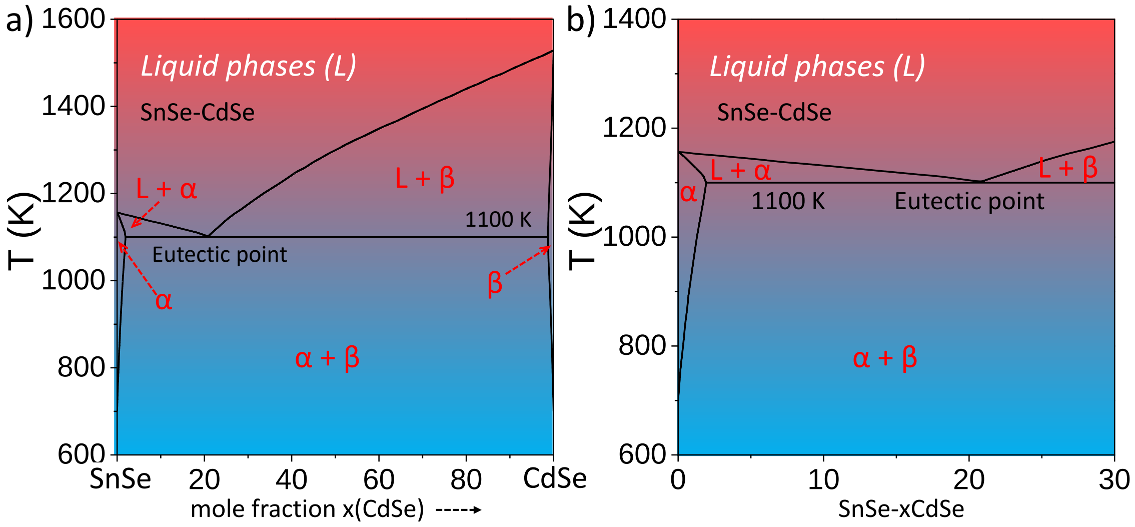

Figure S13. a) Complete SnSe-CdSe phase diagram and b) magnification of the low CdSe content region.[1]

High-temperature XRD analyses of SnSe and SnSe-3%CdSe

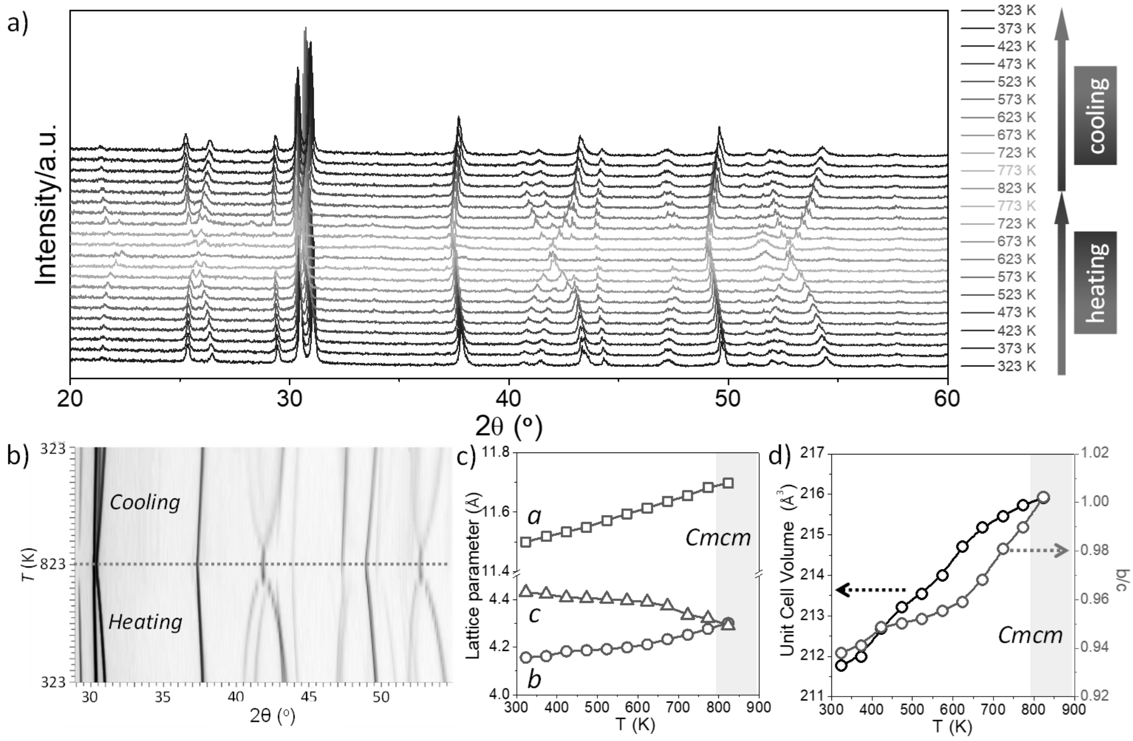

Figure S14. High-temperature XRD analyses of SnSe: a) the evoluation of XRD patterns with the change in temperature from 323 to 823 K followed by subsequent cooling to 323 K; b) 2D plot of intensity as a function of 2θ and T; c) the lattice parameters, and d) lattice parameter ratio b/c and unit cell volume as a function of temperature.

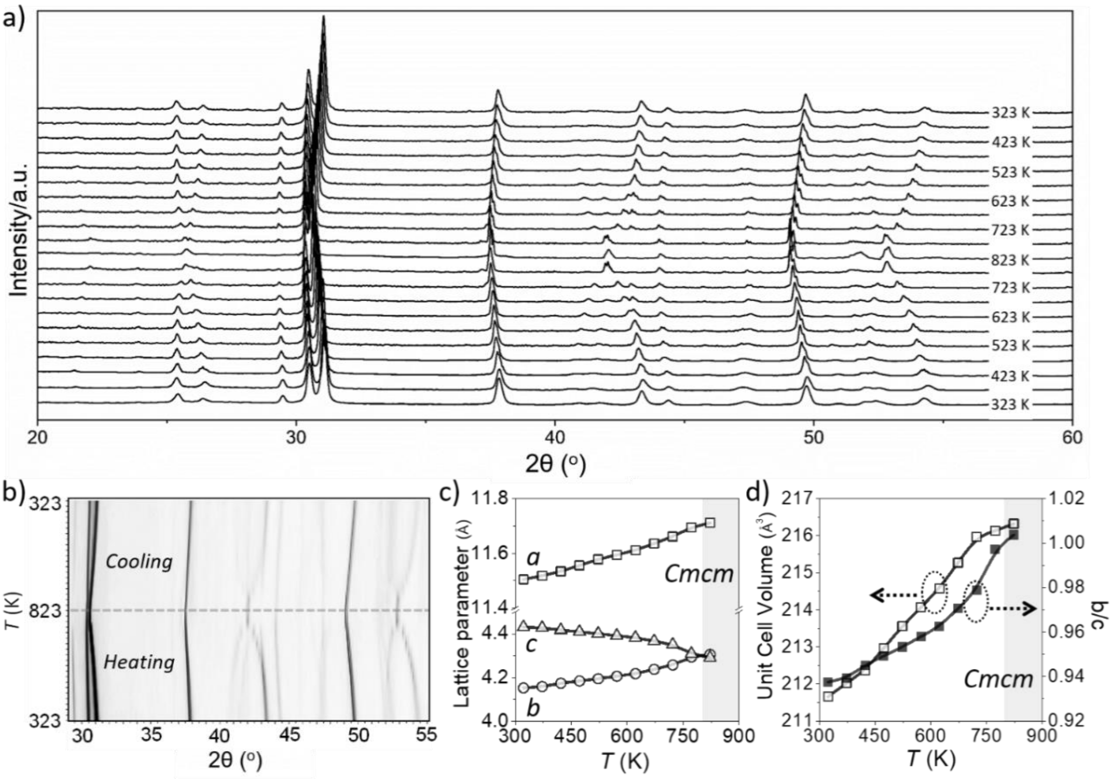

Figure S15. High-temperature XRD analyses of SnSe-3%CdSe nanocomposites: a) the evaluation of XRD patterns with the change in temperature from 323 to 823 K followed by subsequent cooling to 323 K; b) 2D plot of intensity as a function of 2θ and T; c) the lattice parameters, and d) lattice parameter ratio b/c and unit cell volume as a function of temperature.

Lattice parameters and unit cell volume of SnSe-3%PbS pellet

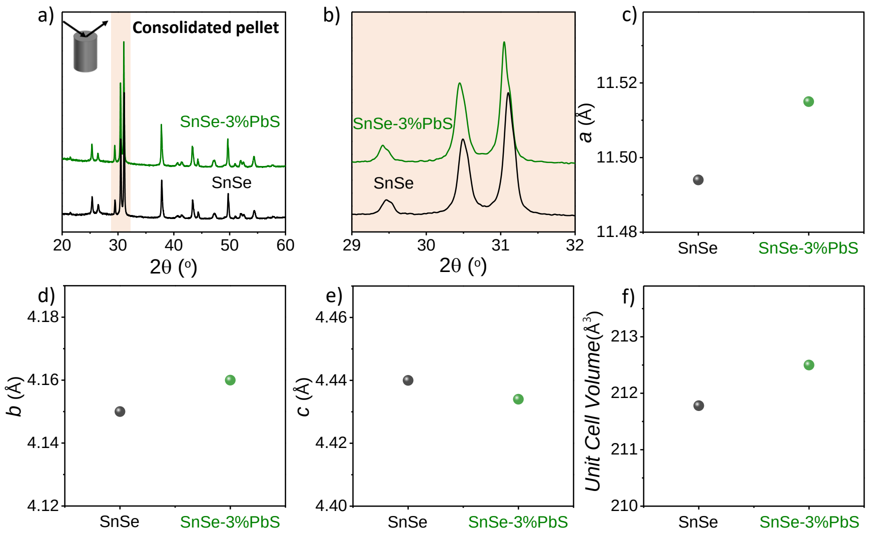

Figure S16. a) XRD patterns of bare SnSe and SnSe-3%PbS pellets in the perpendicular to the pressing axis; b) Magnification of the XRD pattern for the consolidated pellets with and

without PbS surface treatment showing a clear peak shift due to partial alloying of PbS ; c)-f) Experimental lattice parameters and unit cell volume calculated from XRD patterns of bare SnSe and SnSe-3%PbS pellets.

TE properties of SnSe-CdSe samples with different content of CdSe

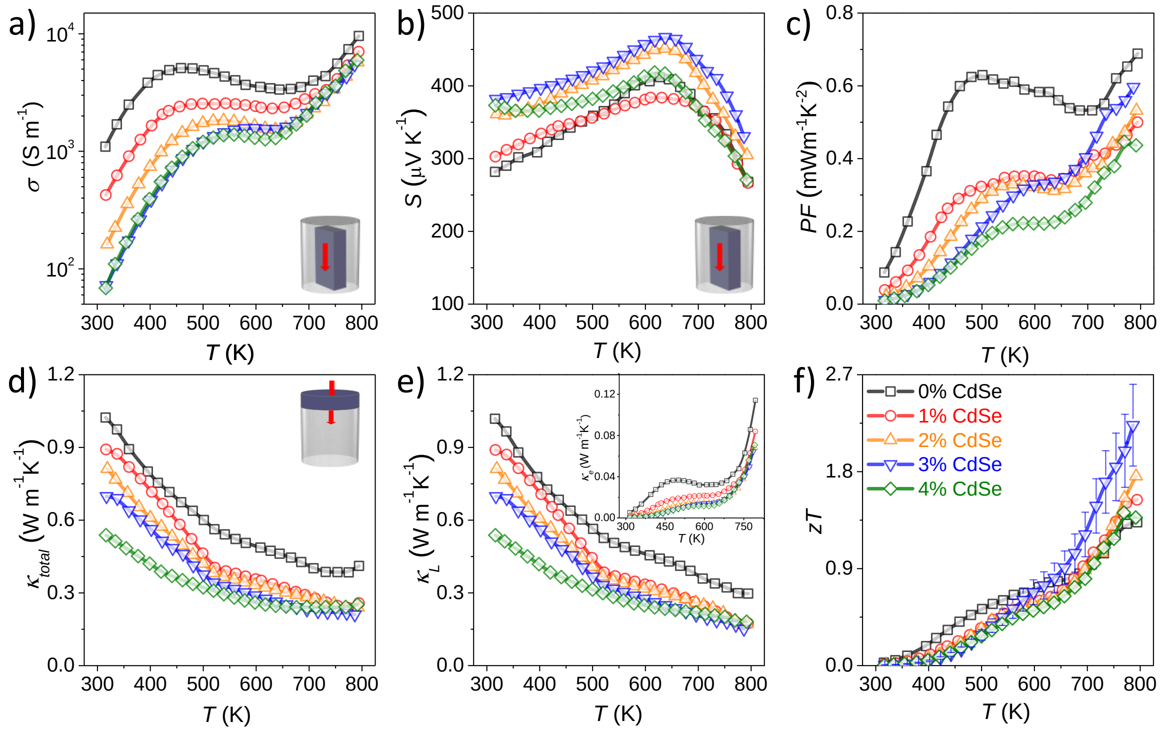

Figure S17. Thermoelectric (TE) properties of SnSe-x%CdSe (x = 0, 1, 2, 3, and 4) samples measured in parallel to the pressing direction: a) electrical conductivity, σ; b) Seebeck coefficient, S; c) power factor, PF; d) thermal conductivity, κ_{total}; e) lattice thermal conductivity, κ_L, electronic thermal conductivity, κ_e (inset); and f) figure-of-merit, zT.

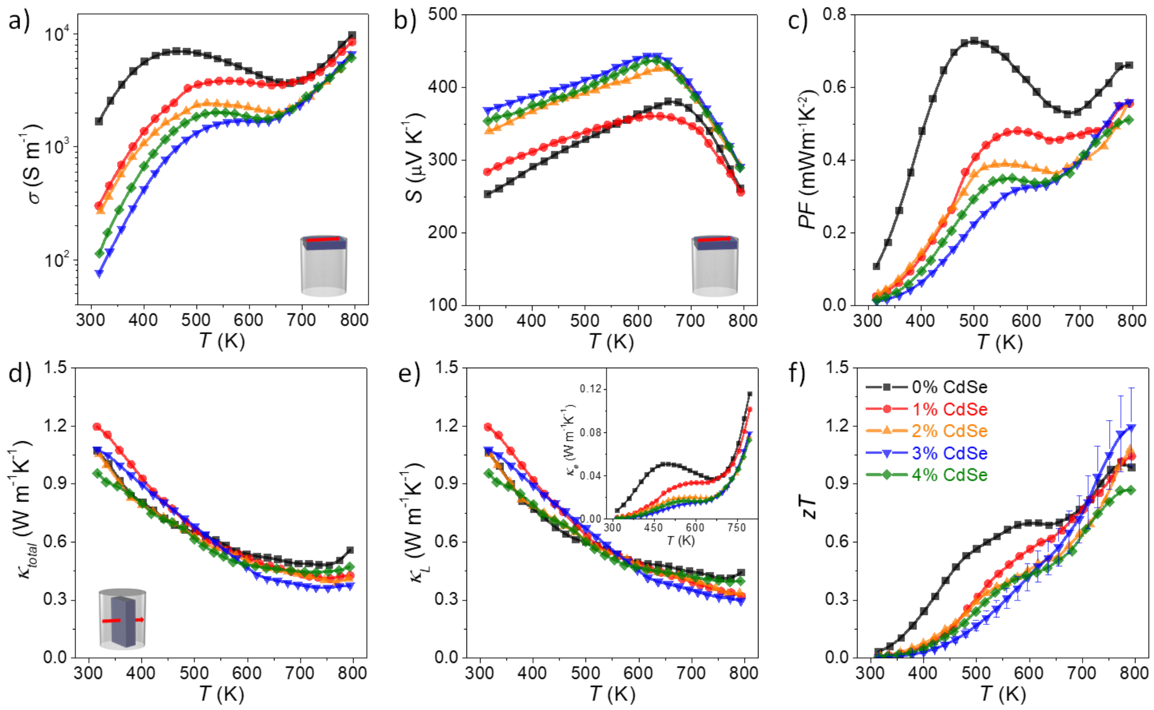

Figure S18. TE properties of bare SnSe and SnSe-x%CdSe (x = 0, 1, 2, 3, and 4) samples measured in perpendicular to the pressing direction: a) electrical conductivity, σ; b) Seebeck coefficient, S; c) power factor, PF; d) thermal conductivity, κ_{total}; e) lattice thermal conductivity, κ_L, electronic thermal conductivity, κ_e (inset); and f) figure-of-merit, zT.

Band structure changes in SnSe induced by the CdSe NPs

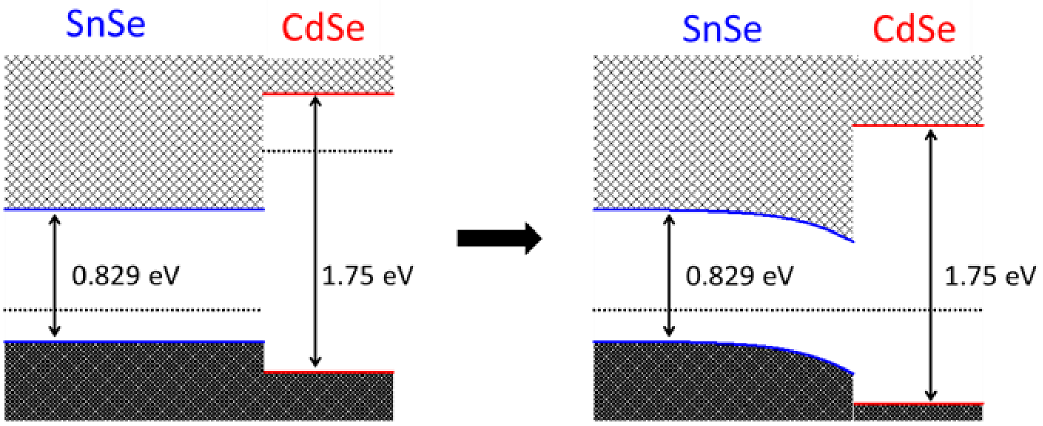

Figure S19. Representation of the band structure changes in SnSe induced by the CdSe NPs in the SnSe/CdSe interface.

TE properties of SnSe and SnSe-3%CdSe measured in parallel direction

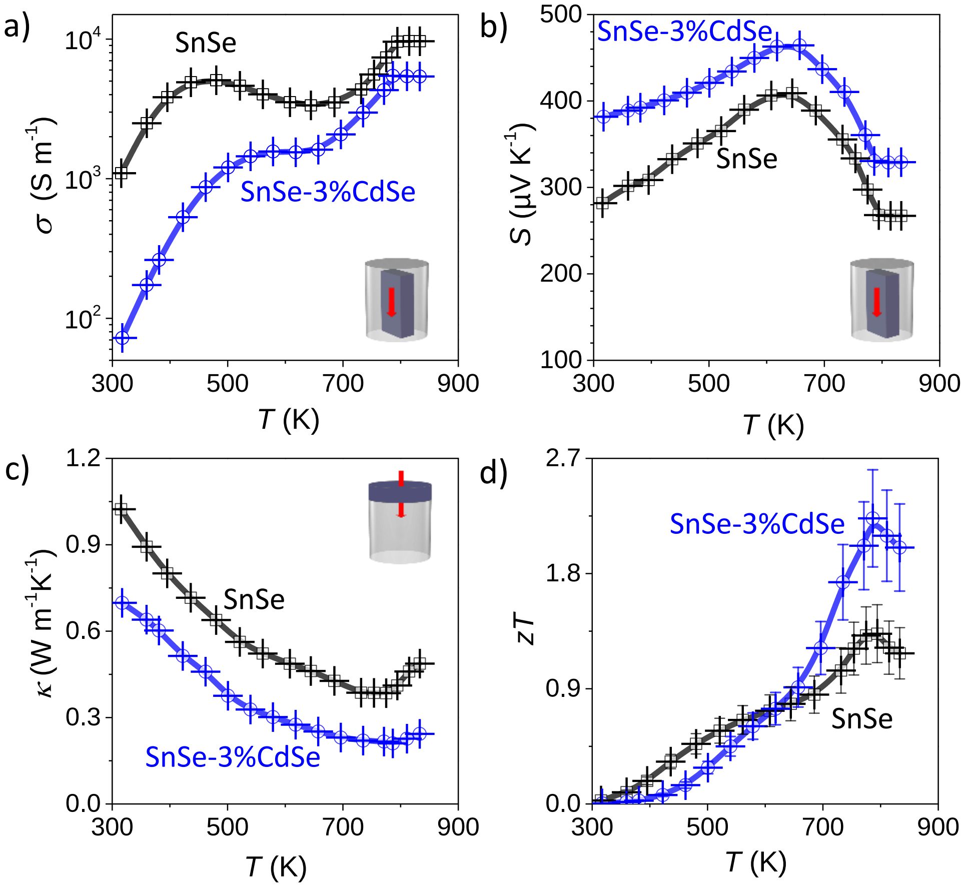

Figure S20. TE properties of bare SnSe and SnSe-3%CdSe samples measured in parallel direction (300 K~833 K): a) electrical conductivity, σ; b) Seebeck coefficient, S; c) thermal conductivity, κ_{total}; and d) figure-of-merit, zT.

Heat capacity C_p of SnSe-3%CdSe

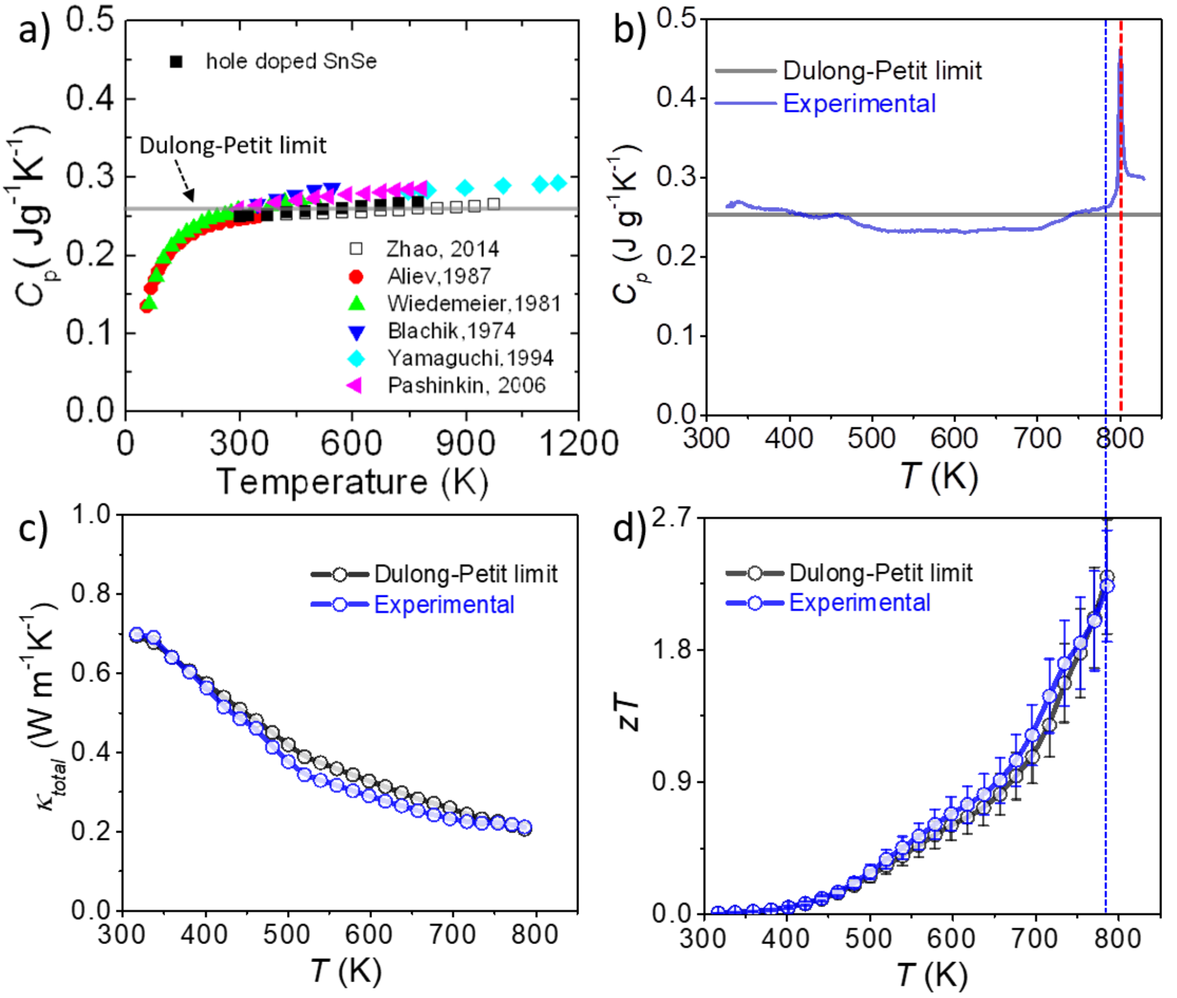

Figure S21. a) The heat capacity C_p of SnSe as a function of temperature. This figure of C_p values is taken from previously reported single crystals and polycrystals by Zhao *et al.*[2] The straight line shows the Dulong-Petit approximation for the C_p. b) The heat capacity C_p calculated from Dulong-Petit approximation (gray) and obtained from experimental C_p values (blue) of SnSe-3%CdSe sample. A comparison of c) total thermal conductivity, κ_{total}; and d) zT calculated from Dulong-Petit approximation and experimental C_p values.

Percentage variations in the TE properties of SnSe-x%CdSe compared to SnSe

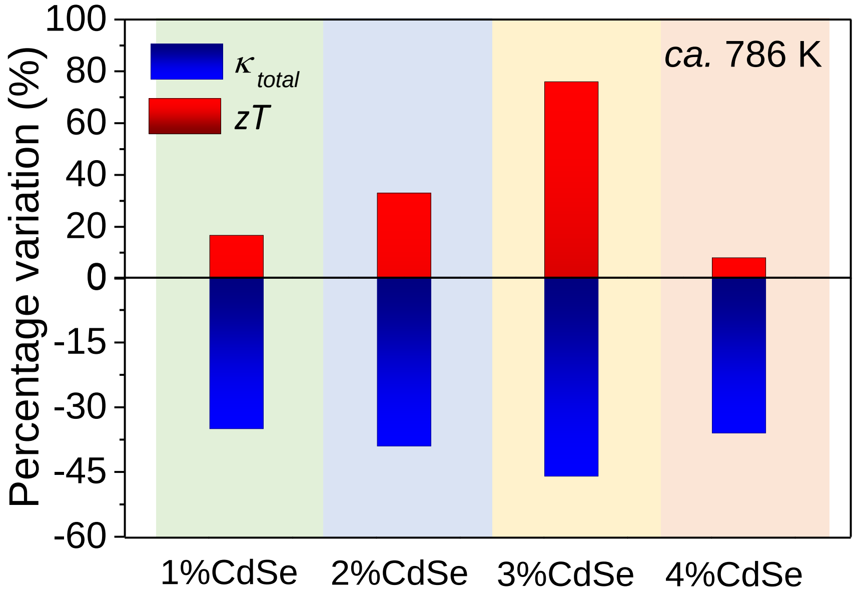

Figure S22. Percentage variations in the TE properties of SnSe-x%CdSe (x = 0, 1, 2, 3, and 4) samples compared to bare SnSe at *ca.* 786 K.

Lattice thermal conductivity (κ_L) calculation

We estimated the lattice thermal conductivity (κ_L) simply by subtracting the electronic thermal conductivity (κ_e) from the measured total thermal conductivity (κ_{total}): $\kappa_L = \kappa_{total} - \kappa_e$.

The electronic contribution κ_e is directly proportional to the electrical conductivity σ according to the Wiedemann-Franz law; $\kappa_e = L\sigma T$, where L is Lorentz number, σ is electrical conductivity and T is the absolute temperature. Here, the Lorenz number L~1.5×10^{-8} V^2K^{-2} is employed in this study, as calculated based on the measured Seebeck coefficient:

$L=[1.5+\exp(-|S|/(116\ \mu VK^{-1}))]\ 10^{-8}\ V^2K^{-2}$

which is proposed by G. J. Snyder *et al*.[3] In fact, $L=1.5\times10^{-8}$ V^2 K^{-2} has been widely used in the literature for SnSe-based materials.[4-8] Figures S17e (inset) and S18e (inset) show the plots of determined temperature-dependent κ_e for SnSe-CdSe nanocomposites, in which the obtained σ showed in Figures S17a and S18a were used for calculating κ_e.

Literature comparison

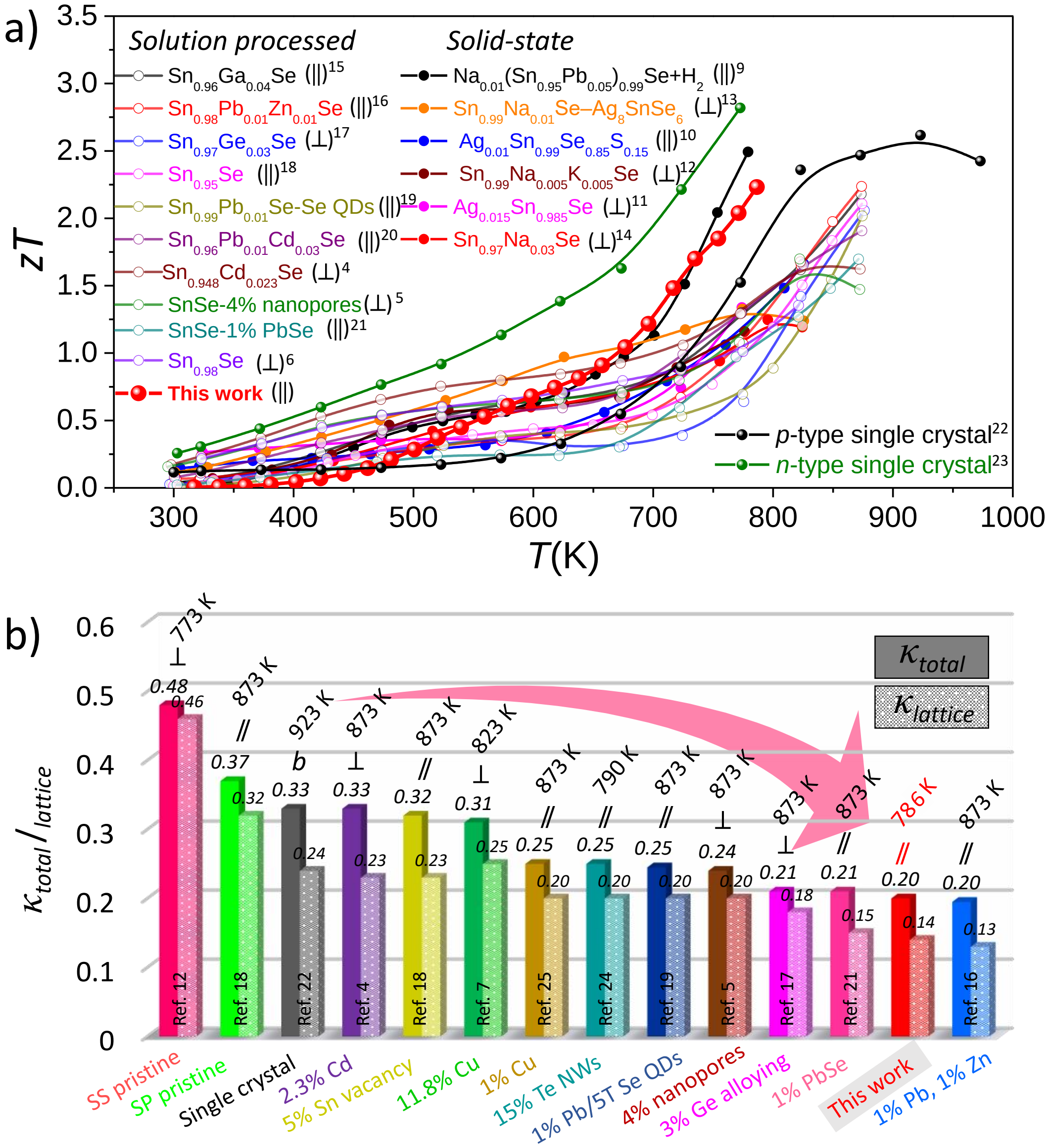

Figure S23. Temperature dependence of zT values for reported state of-the-art p-type polycrystalline SnSe: a) produced by solid-state technology[9-14] and solution-processed technology.[4-6,15-21] The zT values of p-type and n-type single crystals SnSe are also plotted for comparison.[22,23] Results obtained in the present work are plotted with solid red dots and lines (◕). ∥ and ⊥ denotes the direction in which those results were obtained; ∥ refers to parallel and ⊥ refers to perpendicular to SPS pressing direction, respectively. b) Comparison of all the thermal conductivities (κ_{total}) and lattice thermal conductivities (κ_L) for SnSe including p-type single crystal,[22] solid-state (SS) pristine,[12] solution-processed (SP) pristine,[18] 1% Pb-1% Zn-codoped,[16] 3% Ge alloying,[17] 2% Sn vacancy,[18] 1% PbSe,[21] 1% Pb/5T Se QDs,[19] 2.3% Cd,[4] 4% nanopores,[5] 11.8% Cu,[7] 15% Te NWs[24] and 1% Cu[25].

TEM images of SnSe-3%CdSe sample

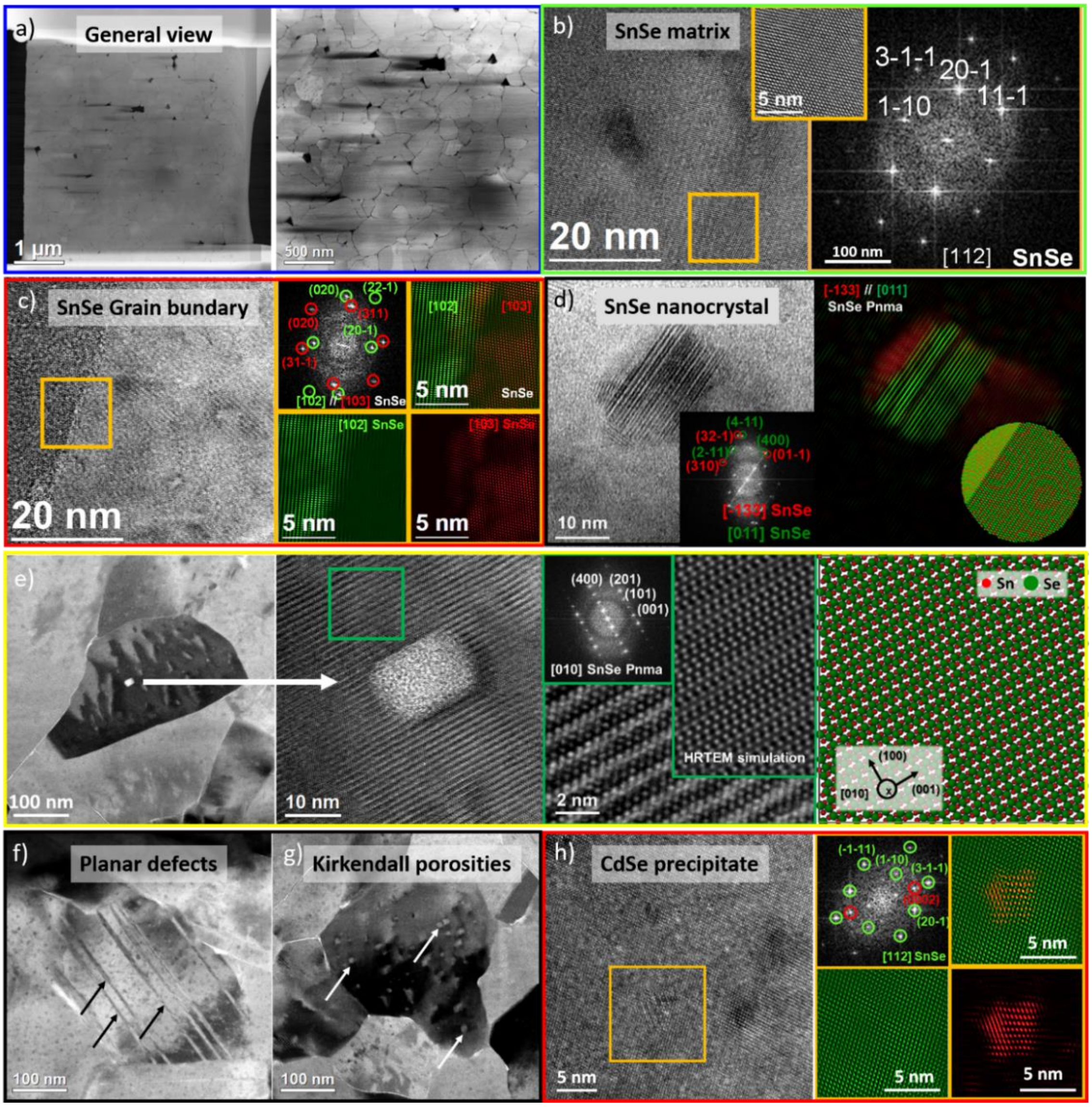

Figure S24. TEM images obtained from the cross-section of the SnSe-3%CdSe nanocomposites. a) General dark-field STEM view of the pellet lamella, where a heterogeneous grain size distribution can be observed; b) HRTEM micrograph of the SnSe matrix, together with their respective indexed power spectra; c) Grain boundary between two SnSe grains; High-resolution transmission electron microscopy images of (d) SnSe Pnma nanocrystal with the corresponding indexed power spectrum (FFT) and phase filtered colored image showing that the nanocrystal is divided into two parts (inset); e) Low magnification image of a large SnSe matrix grain together with two high magnifications views and corresponding power spectrum (inset). The inset also displays the HRTEM image simulation performed on the 3D atomic model reported in the fourth panel; Low-magnification image with enhanced contrast showing a different kind of defect features: f) planar defects and g) porosities; h) HRTEM image of a CdSe precipitate in a *Pnma* SnSe matrix with the corresponding power spectrum

from the region marked in yellow and the phase filtered images corresponding to CdSe precipitate (red) and the SnSe matrix (green).

In Figure S24d, we show a spheroidal SnSe precipitate crystallized in the *Pnma* crystal phase (with *ca.* 20 nm diameter). This precipitate was found in a high symmetry zone axis and it is composed of 2 merged grains (showing a grain boundary). It has a spheroidal morphology, with 2 different sections, both crystallized in the SnSe orthorhombic *Pnma* crystal phase, one oriented along the [011] axis and the other along the [-133]. A 3D atomic model of the complex SnSe precipitate is included in the right panel of Figure S24d. In Figure 7b in the main text, we show an example of a CdSe precipitate with a cubic crystal phase, which is embedded in a SnSe *Pnma* orthorhombic matrix. In Figure S24e, we show an example of SnSe grain presenting a Kinkerdall porosity (see an example in the green squared HRTEM image). If we blow-up in the area squared in green, we can distinguish a peculiar image contrast that is due to the SnSe *Pnma* crystal along the [010] axis. This contrast has been reproduced by performing HRTEM image simulations, as reported in the inset. We performed TEM image simulations by varying defocus conditions and crystal-model thickness (Figure S25), obtaining a good match when the thickness is around 50 nm, which is consistent with typical FIB lamellae thickness.

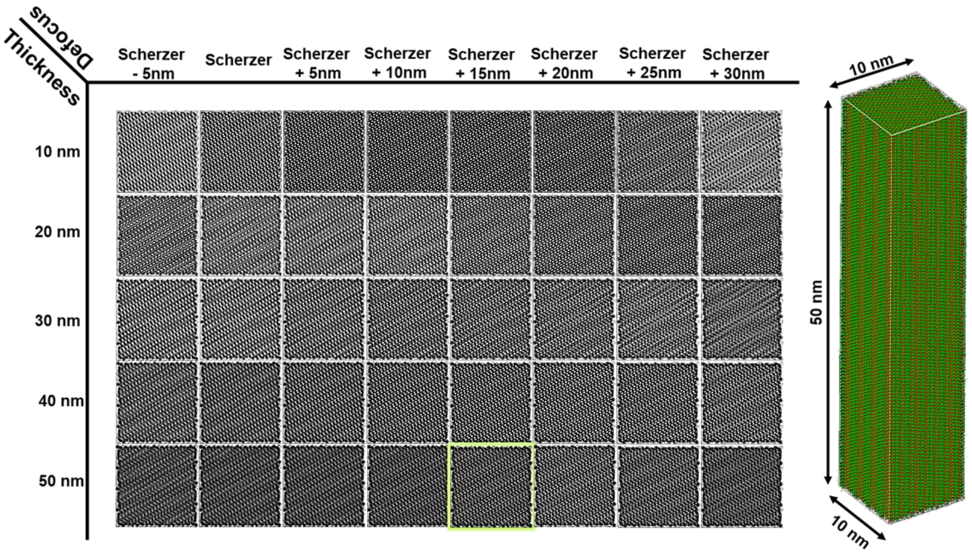

Figure S25. SnSe *Pnma* image simulation as a function of crystal model thickness and defocus condition. The initial 3D atomic model is 10 nm ×10 nm ×50 nm big to reproduce the image contrast reported in Figure 24e. Later the thickness has been reduced with 10 nm steps to investigate thickness related main features.

Material stability and repeatability

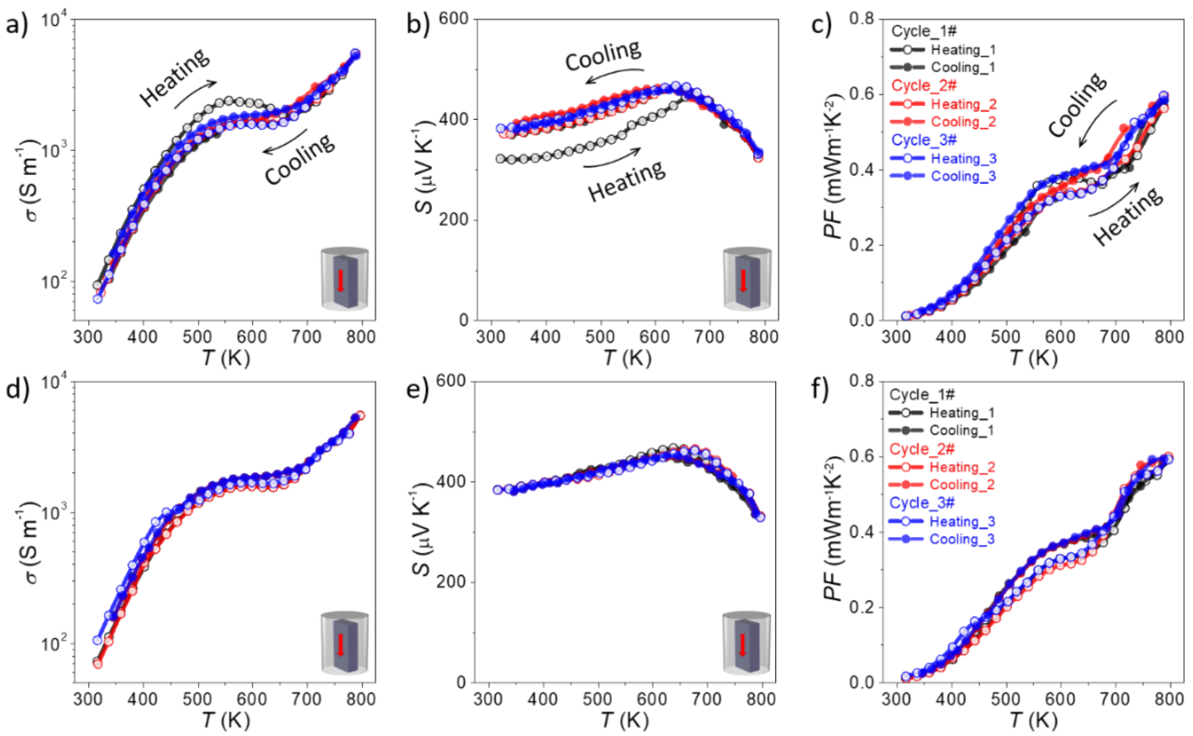

Figure S26. The temperature dependence of (a, d) electrical conductivity (σ), (b, e) Seebeck coefficient (S) and (c, f) power factor (PF) of a SnSe-3%CdSe pellet. a)-c) No pre-stabilization treatment was carried out before the measurements; d)-f) Pellet was annealed in forming gas atmosphere before the measurements. For details, please refer to the section "*bulk nanomaterial consolidation*" in the main text.

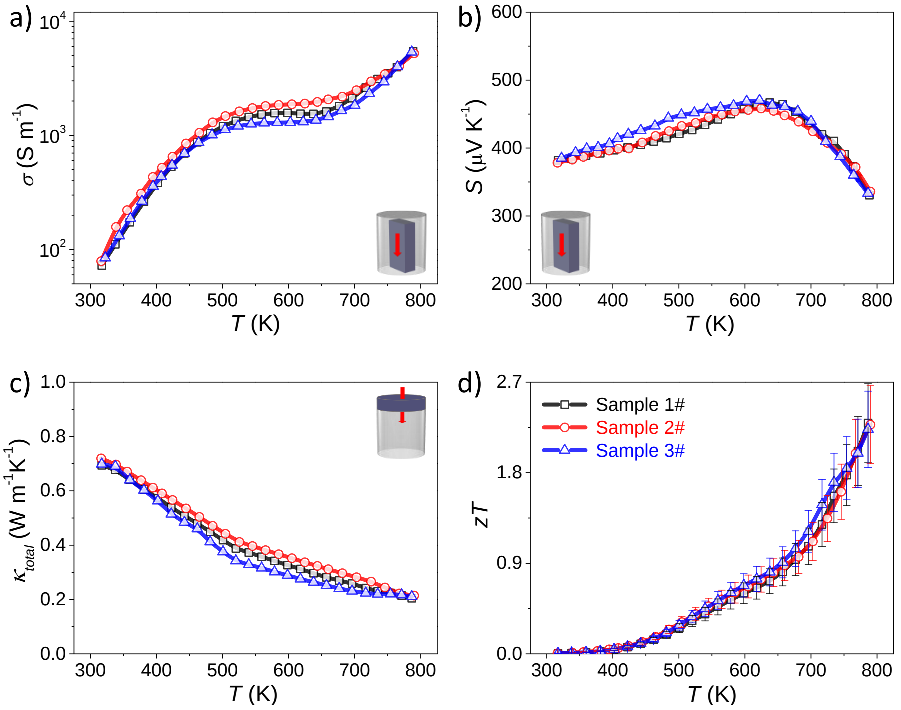

Figure S27. Thermoelectric properties of three SnSe-3%CdSe pellets obtained three different batches: a) electrical conductivity, σ; b) Seebeck coefficient, S; c) thermal conductivity, κ_{total}; and d) figure of merit, zT.

Cylindrical pellet cutting

To measure material transport properties, we cut round shape pellets and rectangular bars, along the pressing direction and within the cylinder plane.

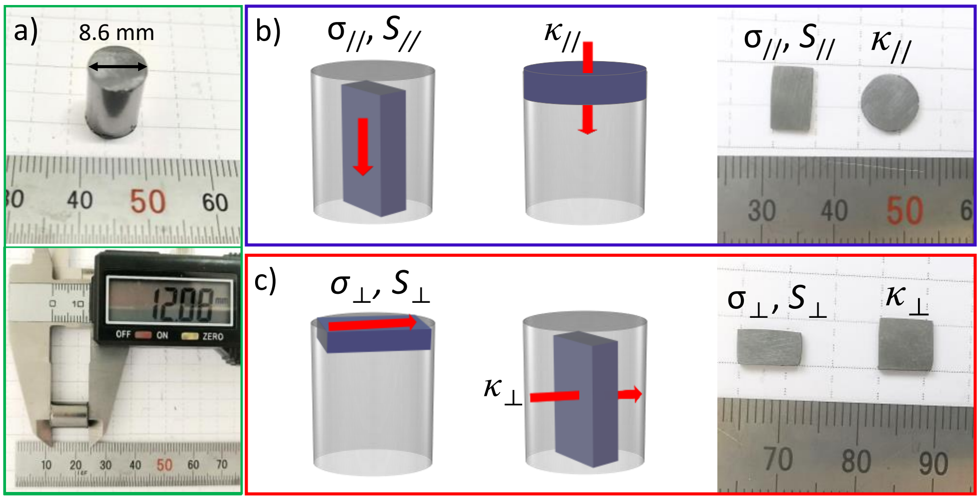

Figure S28. a) Photograph of a cylindrical pellet produced by spark plasma sintering (SPS). (b, c) Schematic illustrations and photographs of the samples obtained from the cylindrical pellets and used to measure TE properties in each direction: parallel b) and perpendicular c) to

the pressing axis. Electrical conductivities (σ) and Seebeck coefficients (S) were always measured using rectangular samples. Thermal conductivities (κ) were measured from disk-shaped samples in the direction of the pressing direction and from rectangular samples in the direction perpendicular to the pressing direction.

Theoretical *zT* prediction

The single parabolic band (SPB) model was employed to calculate the n_H-dependent *zT* values (Figure S29b) at a fixed temperature 750 K (dotted lines) and 786 K (solid lines), and the κ_L used in the calculations are derived from Figure S17e. For the detailed calculations, the carrier transport property analysis was used as:[26,27]

The Seebeck coefficient,

$$S(\eta) = \frac{\kappa_B}{e}\left[\frac{(r+5/2)\cdot F_{r+3/2}(\eta)}{(r+3/2)\cdot F_{r+1/2}(\eta)} - \eta\right] \quad \text{(S1)}$$

The Hall carrier concentration,

$$n_H = \frac{1}{e\cdot R_H} = \frac{(2m^*\cdot \kappa_B T)^{3/2}}{3\pi^2\hbar^3}\cdot\frac{(r+3/2)^2\cdot F_{r+1/2}^2(\eta)}{(2r+3/2)\cdot F_{2r+1/2}(\eta)} \quad \text{(S2)}$$

The Hall mobility,

$$\mu_H = \left[\frac{e\pi\hbar^4}{\sqrt{2}(\kappa_B T)^{3/2}}\frac{C_l}{E_{def}{}^2(m^*)^{5/2}}\right]\frac{(2r+3/2)\cdot F_{2r+1/2}(\eta)}{(r+3/2)^2\cdot F_{r+1/2}(\eta)} \quad \text{(S3)}$$

Lorenz Factor,

$$L = \left(\frac{\kappa_B}{e}\right)^2\left\{\frac{(r+7/2)\cdot F_{r+5/2}(\eta)}{(r+3/2)\cdot F_{r+1/2}(\eta)} - \left[\frac{(r+5/2)\cdot F_{r+3/2}(\eta)}{(r+3/2)\cdot F_{r+1/2}(\eta)}\right]^2\right\} \quad \text{(S4)}$$

Where

$$F_x(\eta) = \int_0^\infty \frac{\varepsilon^x}{1+e^{(\varepsilon-\eta)}}d\varepsilon \quad \text{(S5)}$$

is the Fermi integral.

$$C_l = \upsilon_l^2\rho \quad \text{(S6)}$$

is the deformation potential coefficient.

In the above equations, S, μ_H, η, κ_B, e, r, R_H, $\hbar$, C_l, E_{def}, m^*, L and υ_l are the Seebeck coefficient, the carrier mobility, the reduced Fermi level, the Boltzmann constant, the electron charge, the carrier scattering factor (r=-1/2 for acoustic phonon scattering), the Hall coefficient, the reduced plank constant, the elastic constant for longitudinal vibrations, the deformation potential coefficient, the density of state effective mass, the Lorenz number, and the longitudinal sound velocity,[28] respectively.

We have compared between experimentally achieved figure-of-merit *zTs* with predicted values. The experiment data are close to the calculated result obtained with the SPB model. It is seen that the carrier concentrations $n_H \sim 2.1 \times 10^{19}$ cm^{-3} and $n_H \sim 1.9 \times 10^{19}$ cm^{-3} can contribute to a peak *zT* for SnSe-3% CdSe at 750 K and 786 K, respectively. Here, the predicted values at 786 K were assumed the similar mobility to 750 K and slightly high carrier density according to the tendency of Figure S26a. This indicates that, within the framework of the SPB model, the figure of merit *zT* value can be further improved if the carrier densities are further increased, without degrading the carrier mobility. However, the SPB model in SnSe works well up until hole concentrations of *ca*. 2×10^{19} cm^{-3}. At larger hole concentrations, multi-band transport starts to occur for SnSe and the set of corresponding physics changes similarly. Multi-band transport can enable far superior TE performance than single-band transport and so this is a direction worth pursuing.[29]

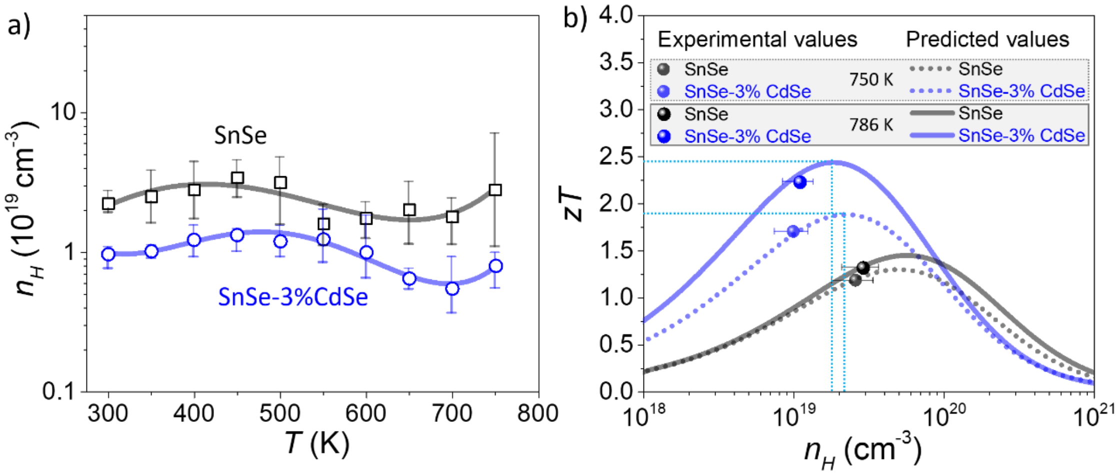

Figure S29. a) Temperature dependence of the Hall charger carrier concentration, n_H; b) n_H-dependent *zT* with both experimental and predicted values at T=750 K and 786 K *via* calculations using the SPB model. The light blue dashed lines indicate the optimal carrier concentration to achieve peak *zT*.

Pellet density and composition

Table S1. Relative densities of SnSe-x%CdSe (x = 0, 1, 2, 3, and 4) pellets obtained from absolute values measured with the Archimedes' method and the theoretical values for SnSe and SnSe-CdSe nanocomposites.

Sample	0	1%	2%	3%	4%
Theoretical density (g/cm^3)	6.18	6.175	6.168	6.164	6.159
Measured density (g/cm^3)	5.73	5.74	5.68	5.69	5.73
Relative density	92.7%	92.9%	92.1%	92.3%	93.0%

Table S2. Composition of SnSe-x%CdSe (x = 0, 1, 2, 3, and 4) pellets obtained from ICP-OES and SEM-EDS analysis (Related to Sn=1). Each data was obtained from averaging five analyses in EDS, resulting in an estimated error of *ca.* 5-10%.

Method	Element atomic ratio	0	1%	2%	3%	4%
ICP-OES	Se	1.08	1.07	1.08	1.08	1.08
	Sn	1	1	1	1	1
	Na	0.016	0.016	0.016	0.016	0.016
	Cd	0	0.008	0.019	0.028	0.039
SEM-EDS	Se	1.06	1.06	1.07	1.06	1.07
	Sn	1	1	1	1	1
	Na	0.015	0.016	0.015	0.015	0.016
	Cd	0	0.010	0.021	0.031	0.041

References

(1) Leute, V.; Menge, D. Thermodynamic Investigations of the Quasibinary Systems $(Cd_KSn_{1-K})Te$, $(Cd_KSn_{1-K})Se$, And $Sn(Se_LTe_{1-L})$. *Zeitschrift fur Phys. Chemie* **1992**, *176*, 47–64.

(2) Zhao, L.-D.; Tan, G.; Hao, S.; He, J.; Pei, Y.; Chi, H.; Wang, H.; Gong, S.; Xu, H.; Dravid, V. P.; Uher, C.; Snyder, G. J.; Wolverton, C.; Kanatzidis, M. G. Ultrahigh Power Factor and Thermoelectric Performance in Hole-Doped Single-Crystal SnSe. *Science* **2016**, *351*, 141–144.

(3) Kim, H. S.; Gibbs, Z. M.; Tang, Y.; Wang, H.; Snyder, G. J. Characterization of Lorenz Number with Seebeck Coefficient Measurement. *APL Mater.* **2015**, *3*, 1–6.

(4) Shi, X.; Wu, A.; Feng, T.; Zheng, K.; Liu, W.; Sun, Q.; Hong, M.; Pantelides, S. T.; Chen, Z.-G.; Zou, J. High Thermoelectric Performance in *p*-Type Polycrystalline Cd-Doped SnSe Achieved by a Combination of Cation Vacancies and Localized Lattice Engineering. *Adv. Energy Mater.* **2019**, *9*, 1803242.

(5) Shi, X.; Wu, A.; Liu, W.; Moshwan, R.; Wang, Y.; Chen, Z.-G.; Zou, J. Polycrystalline SnSe with Extraordinary Thermoelectric Property *via* Nanoporous Design. *ACS Nano* **2018**, *12*, 11417–11425.

(6) Shi, X.; Chen, Z. G.; Liu, W.; Yang, L.; Hong, M.; Moshwan, R.; Huang, L.; Zou, J. Achieving High Figure of Merit in *p*-Type Polycrystalline $Sn_{0.98}Se$ *via* Self-Doping and Anisotropy-Strengthening. *Energy Storage Mater.* **2018**, *10*, 130–138.

(7) Shi, X.; Zheng, K.; Hong, M.; Liu, W.; Moshwan, R.; Wang, Y.; Qu, X.; Chen, Z. G.; Zou, J. Boosting the Thermoelectric Performance of *p*-Type Heavily Cu-Doped Polycrystalline SnSe: *Via* Inducing Intensive Crystal Imperfections and Defect Phonon Scattering. *Chem. Sci.* **2018**, *9*, 7376–7389.

(8) Hong, M.; Chen, Z. G.; Yang, L.; Chasapis, T. C.; Kang, S. D.; Zou, Y.; Auchterlonie, G. J.; Kanatzidis, M. G.; Snyder, G. J.; Zou, J. Enhancing the Thermoelectric Performance of $SnSe_{1-x}Te_x$ Nanoplates through Band Engineering. *J. Mater. Chem. A* **2017**, *5*, 10713–10721.

(9) Lee, Y. K.; Luo, Z.; Cho, S. P.; Kanatzidis, M. G.; Chung, I. Surface Oxide Removal for Polycrystalline SnSe Reveals Near-Single-Crystal Thermoelectric Performance. *Joule* **2019**, *3*, 719–731.

(10) Lin, C.-C.; Lydia, R.; Yun, J. H.; Lee, H. S.; Rhyee, J. S. Extremely Low Lattice Thermal Conductivity and Point Defect Scattering of Phonons in Ag-Doped $(SnSe)_{1-x}(SnS)_x$ Compounds. *Chem. Mater.* **2017**, *29*, 5344–5352.

(11) Zhang, L.; Wang, J.; Sun, Q.; Qin, P.; Cheng, Z.; Ge, Z.; Li, Z.; Dou, S. Three-Stage Inter-Orthorhombic Evolution and High Thermoelectric Performance in Ag-Doped Nanolaminar SnSe Polycrystals. *Adv. Energy Mater.* **2017**, *7*, 1700573.

(12) Ge, Z.-H.; Song, D.; Chong, X.; Zheng, F.; Jin, L.; Qian, X.; Zheng, L.; Dunin-Borkowski, R. E.; Qin, P.; Feng, J.; Zhao, L. -D. Boosting the Thermoelectric Performance of (Na,K)-Codoped Polycrystalline SnSe by Synergistic Tailoring of the Band Structure and Atomic-Scale Defect Phonon Scattering. *J. Am. Chem. Soc.* **2017**, *139*, 9714–9720.

(13) Luo, Y.; Cai, S.; Hua, X.; Chen, H.; Liang, Q.; Du, C.; Zheng, Y.; Shen, J.; Xu, J.;

Wolverton, C.; Dravid, V. P.; Yan, Q.; Kanatzidis, M. G. High Thermoelectric Performance in Polycrystalline SnSe *via* Dual-Doping with Ag/Na and Nanostructuring with Ag_8SnSe_6. *Adv. Energy Mater.* **2019**, *9*, 1803072.

(14) Liang, S.; Xu, J.; Noudem, J. G.; Wang, H.; Tan, X.; Liu, G.-Q.; Shao, H.; Yu, B.; Yue, S.; Jiang, J. Thermoelectric Properties of Textured Polycrystalline $Na_{0.03}Sn_{0.97}Se$ Enhanced by Hot Deformation. *J. Mater. Chem. A* **2018**, *6*, 23730–23735.

(15) Lou, X.; Li, S.; Chen, X.; Zhang, Q.; Deng, H.; Zhang, J.; Li, D.; Zhang, X.; Zhang, Y.; Zeng, H.; Tang, G. Lattice Strain Leads to High Thermoelectric Performance in Polycrystalline SnSe. *ACS Nano* **2021**. *15*, 8204-8215.

(16) Liu, J.; Wang, P.; Wang, M.; Xu, R.; Zhang, J.; Liu, J.; Li, D.; Liang, N.; Du, Y.; Chen, G.; Tang, G. Achieving High Thermoelectric Performance with Pb and Zn Codoped Polycrystalline SnSe *via* Phase Separation and Nanostructuring Strategies. *Nano Energy* **2018**, *53*, 683–689.

(17) Chandra, S.; Biswas, K. Realization of High Thermoelectric Figure of Merit in Solution Synthesized 2D SnSe Nanoplates *via* Ge Alloying. *J. Am. Chem. Soc.* **2019**, *141*, 6141–6145.

(18) Wei, W.; Chang, C.; Yang, T.; Liu, J.; Tang, H.; Zhang, J.; Li, Y.; Xu, F.; Zhang, Z.; Li, J.-F.; Tang, G. Achieving High Thermoelectric Figure of Merit in Polycrystalline SnSe *via* Introducing Sn Vacancies. *J. Am. Chem. Soc.* **2018**, *140*, 499–505.

(19) Xu, R.; Huang, L.; Zhang, J.; Li, D.; Liu, J. J.; Liu, J. J.; Fang, J.; Wang, M.; Tang, G. Nanostructured SnSe Integrated with Se Quantum Dots with Ultrahigh Power Factor and Thermoelectric Performance from Magnetic Field-Assisted Hydrothermal Synthesis. *J. Mater. Chem. A* **2019**, *7*, 15757–15765.

(20) Li, S.; Lou, X.; Li, X.; Zhang, J.; Li, D.; Deng, H.; Liu, J.; Tang, G. Realization of High Thermoelectric Performance in Polycrystalline Tin Selenide through Schottky Vacancies and Endotaxial Nanostructuring. *Chem. Mater.* **2020**, *32*, 9761–9770.

(21) Tang, G.; Wei, W.; Zhang, J.; Li, Y.; Wang, X.; Xu, G.; Chang, C.; Wang, Z.; Du, Y.; Zhao, L.-D. Realizing High Figure of Merit in Phase-Separated Polycrystalline $Sn_{1-x}Pb_xSe$. **2016**, *138*, 13647–13654.

(22) Zhao, L.-D.; Lo, S.-H.; Zhang, Y.; Sun, H.; Tan, G.; Uher, C.; Wolverton, C.; Dravid, V. P.; Kanatzidis, M. G. Ultralow Thermal Conductivity and High Thermoelectric Figure of Merit in SnSe Crystals. *Nature* **2014**, *508*, 373–377.

(23) Chang, C.; Wu, M.; He, D.; Pei, Y.; Wu, C.-F. F.; Wu, X.; Yu, H.; Zhu, F.; Wang, K.; Chen, Y.; Huang, L.; Li, J. F.; He, J.; Zhao, L. -D. 3D Charge and 2D Phonon Transports Leading to High Out-of-Plane ZT in *n*-Type SnSe Crystals. *Science* **2018**, *360*, 778–783.

(24) Li, M.; Liu, Y.; Zhang, Y.; Zuo, Y.; Li, J.; Lim, K. H.; Cadavid, D.; Ng, K. M.; Cabot, A. Crystallographically Textured SnSe Nanomaterials Produced from the Liquid Phase Sintering of Nanocrystals. *Dalt. Trans.* **2019**, *48*, 3641–3647.

(25) Gong, Y.; Chang, C.; Wei, W.; Liu, J.; Xiong, W.; Chai, S.; Li, D.; Zhang, J.; Tang, G. Extremely Low Thermal Conductivity and Enhanced Thermoelectric Performance of Polycrystalline SnSe by Cu Doping. *Scr. Mater.* **2018**, *147*, 74–78.

(26) Shen, J.; Chen, Z.; Lin, S.; Zheng, L.; Li, W.; Pei, Y. Single Parabolic Band Behavior of Thermoelectric *p*-Type $CuGaTe_2$. *J. Mater. Chem. C* **2015**, *4*, 209–214.

(27) Goldsmid, H. J. *Introduction to Thermoelectricity*; Springer: Berlin, 2010.

(28) Xiao, Y.; Chang, C.; Pei, Y.; Wu, D.; Peng, K.; Zhou, X.; Gong, S.; He, J.; Zhang, Y.; Zeng, Z.; Zhao, L.-D. Origin of Low Thermal Conductivity in SnSe. *Phys. Rev. B* **2016**, *94*, 125203.

(29) Chang, C.; Tan, G.; He, J.; Kanatzidis, M. G.; Zhao, L.-D. The Thermoelectric Properties of SnSe Continue to Surprise: Extraordinary Electron and Phonon Transport. *Chem. Mater.* **2018**, *30*, 7355–7367.
